# Design and Performance Evaluation of Unpowered Hip-Assisted Exoskeletons

**DOI:** 10.3390/biomimetics11090625

**Published:** 2026-09-02

**Authors:** Xinyao Tang, Xupeng Wang, Xinying Xue, Hongyan Liu, Mengyuan Pu

**Affiliations:** 1 Research Center for Civil-Military Integration and Protection Equipment Design Innovation, Xi’an University of Technology, Xi’an 710054, China; 2 Digital Intelligence Innovation Design Department, Xi’an University of Technology, Xi’an 710054, China

**Keywords:** unpowered exoskeleton, hip assistance, comprehensive evaluation method, gait analysis, electromyography, metabolic energy consumption

## Abstract

Human augmentation is an important branch of robotics research aimed at reducing metabolic energy consumption, delaying fatigue, and increasing body speed. However, existing evaluation protocols lack systematic frameworks for unpowered hip devices. This study aims to reduce the energy consumption of human movement without providing additional power and to develop a hip-assisted exoskeleton device. Through gait, plantar pressure, and electromyography tests, the system studied the assistance performance of exoskeletons in three wearing states: “No exo.”, “Exo. on”, and “Exo. off”. A comprehensive evaluation method of unpowered lower limb wearable exoskeleton (CE-ULLWE) is established, featuring the novel three-condition design that isolates the structural mass effect from true assistance via the “Exo. off” condition, and integrates multi-indicator metrics including kinematics, dynamics, plantar pressure, EMG, and metabolic simulations. Combining wearing and exercise testing to obtain the human–machine compatibility of exoskeletons and the subjective comfort of users when wearing exoskeletons. Experimental results demonstrate that wearing the exoskeleton increases peak hip and knee angular velocities by 18.5% and 9.0%, reduces joint power, decreases plantar pressure center excursion by 32.3%, and lowers total metabolic energy consumption by 16.0%, confirming its effectiveness in reducing metabolic cost and delaying fatigue. This achievement has important theoretical guidance and practical application value for the design, function, and performance evaluation of wearable assistive exoskeleton products. The proposed CE-ULLWE offers a replicable, multi-indicator framework that clarifies assistive efficacy and guides future exoskeleton optimization.

## 1. Introduction

As a basic form of daily activities, human walking and running not only directly affect the quality of life, but also play a key role in rehabilitation, auxiliary and industrial scenes. However, with the increase of age, nerve injury or limb dysfunction, individuals often face problems such as the decline of exercise ability and the increase of metabolic energy consumption, which lead to the limitation of daily activities and the decline of quality of life. Although traditional assistive devices such as wheelchairs provide support, they have limitations such as lack of flexibility and muscle atrophy caused by long-term use [1]. As a new solution, exoskeleton technology has become a hot spot in the field of biomedical engineering to enhance human movement ability through mechanical structure, and advanced sliding mode anti-disturbance control strategies have been widely adopted to guarantee stable gait tracking for rehabilitation exoskeleton actuators and knee joint robotic systems under complex external interferences [2]. The motor-driven lower limb exoskeleton adapts flexibly to gait assistance, but its complexity and energy cost are high, and distributed adaptive control provides an anti-interference reference for this driving system [3]. Among them, hip joint-assisted exoskeletons are particularly important in lower limb movements, as the hip joint contributes the majority of energy transfer and stability in gait. Compared with the active drive system, a passive or unpowered exoskeleton provides a sustainable power assistance scheme with its advantages of lightweight, low cost and freedom from external energy dependence [4]. This design recovers negative mechanical energy by storing mechanical energy in a specific gait stage and releasing it in the acceleration stage, so as to reduce metabolic energy consumption and optimize gait efficiency [5].

Despite the potential of the unpowered hip exoskeleton, its design and performance evaluation still face major challenges. First of all, there are significant biomechanical differences between walking and running. Walking involves symmetrical gait and stable support. Running requires high-speed dynamic balance, while the traditional exoskeleton is difficult to adapt to the two gait modes synchronously, resulting in increased equipment weight and metabolic energy penalty [6]. Secondly, the mechanical compatibility between the exoskeleton and the human body is often limited; the joint center is not aligned or the rigid structure limits the range of motion, causing user discomfort or abnormal gait [7]. Design challenges also include weight control; for example, an overweight exoskeleton will increase the metabolic burden of users and may even offset the auxiliary effect it provides [8], as well as the reliability and energy storage efficiency of coil springs or pneumatic mechanisms [5,9]. In terms of performance evaluation, the existing methods focus on quantitative indicators such as metabolic rate reduction, but ignore the dynamic stability analysis of gait stage, which may lead to incomplete evaluation results [10]. In addition, personalized needs in clinical rehabilitation applications, such as gait differences in stroke patients, require real-time and adaptive assessment strategies [11,12].

In the field of unpowered design, the unpowered hip exoskeleton developed by Zhou et al. reduces the metabolic rate of walking and running through the mechanical energy storage mechanism. Its advantage is that it does not need motor drive and directly reduces the system complexity [6]. Similarly, the passive hip exoskeleton designed by Hu et al. uses a double stable switch and a coil spring to store negative energy in the deceleration phase and use it in the acceleration phase to improve walking efficiency [5]. Although these designs are innovative, the response speed and energy conversion efficiency of the storage mechanism at high speed still need to be optimized. Another kind of quasi-passive design, such as a pneumatic system, integrates soft materials and elastic components to improve wearability, but relying on an air pressure source limits its purely non-dynamic characteristics [9,13]. In terms of evaluation methods, the new trend turns to multi-dimensional analysis. For example, Gonabadi et al. [10] introduced the gait tube method to visualize the velocity trajectory of the center of gravity to evaluate the phase-dependent stability when wearing an exoskeleton, revealing the difference in the influence of assistance on different gait stages. The performance index was extended from simple metabolic reduction to gait kinematics parameters. Lenzi et al. evaluated that the residual hip effort of a light dynamic hip exoskeleton in knee amputees was reduced without affecting the kinematic balance [14]. However, the limitations of the existing evaluation methods lie in the small sample size and the lack of cross-population validation. For example, Li’s randomized trial only included 40 stroke patients [11].

In short, wearable robots have developed rapidly in the past few years. It is very important to quantify the effectiveness of wearable exoskeleton performance and turn it into a practical benchmark. The comprehensive performance evaluation is an objective measure of the pros and cons of exoskeleton equipment, which involves many factors. The main contributions of this study are as follows:

(1) A comprehensive evaluation of the unpowered lower limb wearable exoskeleton (CE-ULLWE) method is proposed, focusing on human power assistance through five systematic steps including human–machine compatibility test, phased experimental tasks, performance index evaluation, experimental data processing and statistical analysis;
(2) Eight core performance indicators (PI) are established across gait variables, plantar pressure variables and physiological variables to comprehensively quantify the assistive effectiveness of the unpowered hip exoskeleton;
(3) The proposed evaluation framework is experimentally validated through gait, plantar pressure, and electromyography tests under three wearing conditions (“No exo.”, “Exo. on”, and “Exo. off”), enabling the isolation of the mass effect of the exoskeleton from its actual assistive performance.

The remainder of this paper is organized as follows. Section 2 elaborates on the exoskeleton modeling development, covering the energy storage unit matching test and the mechanical design of the hip-assisted exoskeleton. Section 3 details the CE-ULLWE method, including human–machine compatibility, experimental tasks, performance indicators, data processing, and statistical procedures. Section 4 presents and discusses the results from gait, plantar pressure, and electromyography tests under three wearing conditions. Section 5 summarizes the conclusions and highlights the main contributions.

## 2. Development of Hip Exoskeleton

### 2.1. Matching Testing of Energy Storage Unit

**Figure 1:**
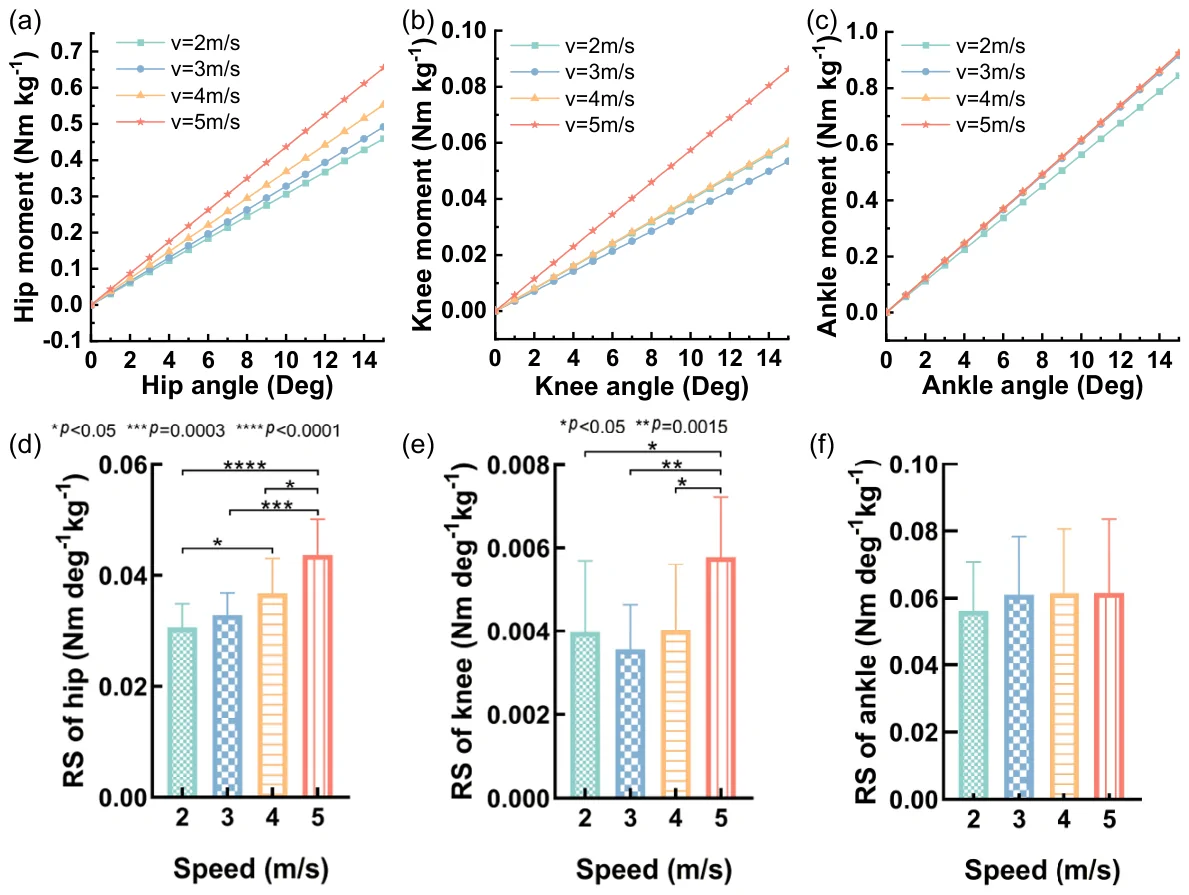
Joint moment—angle curves: (**a**) hip, (**b**) knee, and (**c**) ankle. Comparison with RS: (**d**) hip, (**e**) knee, and (**f**) ankle.

The stiffness parameters of the elastic energy storage component determine the auxiliary torque output characteristics of the unpowered hip exoskeleton, directly affecting the coordination comfort and metabolic energy-saving efficiency of the human–machine, making it a core design parameter that must be clearly defined before prototype development. Previous studies on passive exoskeletons mostly used fixed spring stiffness without considering gait differences at different motion speeds, which can easily lead to a mismatch in auxiliary torque between mechanical structures and human biological joint torque. Excessive spring stiffness can bring additional resistance during walking, increasing the burden on lower limb muscles, while insufficient stiffness cannot store enough negative mechanical energy to generate effective auxiliary force. To avoid this design flaw, the gait data of 10 experimenters running at 2/3/4/5 m/s were collected. The average age, height and weight of the experimenters were 29 ± 5 years old, 1.77 ± 0.04 m and 70.9 ± 7.0 kg, respectively. The inverse kinematics and dynamics were simulated by OpenSim to analyze the characteristics of joint moment and angle, as shown in Figure 1. By quantitatively analyzing these curves, the distribution of rotational stiffness of the human hip, knee, and ankle joints under variable motion speeds was analyzed, providing an objective biomechanical basis for the preload and stiffness matching of the self-developed hip exoskeleton variable stiffness energy storage unit.

Rotational stiffness (RS) is defined as the ratio of joint moment to joint angle. According to the motion capture data at different motion speeds, the variation characteristics of joint rotational stiffness at different motion speeds were explored. As shown in Figure 1a–c, the moment of the hip, knee and ankle varies linearly with the joint rotation angle at different motion speeds, and the slope of the straight line represents the rotational stiffness of each joint at different motion speeds. As shown in Figure 1d–f, the comparative analysis of rotational stiffness of hip, knee and ankle joints under different motion speeds is shown in a box diagram.

Through the comparative analysis of the simulation results, it is found that the rotational moment of the hip increases significantly with the increase in motion speed, and the rotational moment of the knee is significantly affected by the change in motion speed, but the rotational stiffness of the ankle is hardly affected by the four kinds of motion speed, and there is no significant change. The simulation results show that: (1) the rotational stiffness of the hip in 2 m/s motion state is significantly lower than that in 4 m/s and 5 m/s motion state; (2) The rotational stiffness of hip and knee in 3 m/s motion state had no significant relationship with 2 m/s and 4 m/s motion state; (3) When moving at 5 m/s, the rotational stiffness of hip and knee is significantly increased compared with the rest of the speed. Therefore, when designing the assisted exoskeleton, the appropriate assistance should be selected according to the change of rotational stiffness in 2 m/s and 5 m/s motion states, respectively. Only by selecting the appropriate assistance according to different motion speeds can a better auxiliary effect be achieved.

The above simulation results show that the motion speed will affect the rotational stiffness of the joint, so the appropriate assistance should be selected according to different motion speeds. From the perspective of human sports biomechanics, the selection of optimal assistance should be able to minimize the cost of human sports energy consumption. Therefore, the rotational stiffness at a walking speed of 2 m/s was selected as the research objective to study the effect of different auxiliary coefficients on the metabolic curve.

### 2.2. Design of Energy Storage Spring Stiffness

**Figure 2:**
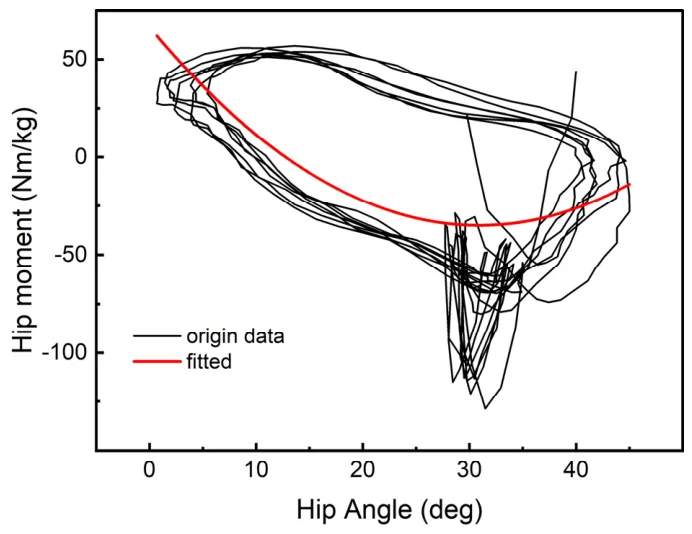
Hip joint stiffness fitting curves.

Based on the analysis results in the previous section, the average values of joint torque and angle data at a speed of 2 m/s were selected. The joint torque angle diagram was drawn and subjected to nonlinear fitting, as shown in Figure 2.

In order to find the best assistance and lay a theoretical foundation for the development of the assisted exoskeleton, it is necessary to obtain the correlation between the assisted exoskeleton torque and the biological movement torque. Since R can also be estimated by the standard score mean of sample points, the auxiliary coefficient $k_{joi}$ is proposed, and its relationship with the assisted $T_{ass}$ and biological torque $T_{bio}$ is as follows,

(1) $$T_{ass}=k_{joi}\times T_{bio}$$

where the values of $k_{joi}$ = 0 or 1 represent no assisted and completely assisted, respectively. Therefore, in order to achieve the effect of variable stiffness assistance, this study uses spiral springs with fixed stiffness properties and pagoda-shaped springs with variable stiffness properties as the core energy storage units.

(1) Spiral spring selection

**Figure 3:**
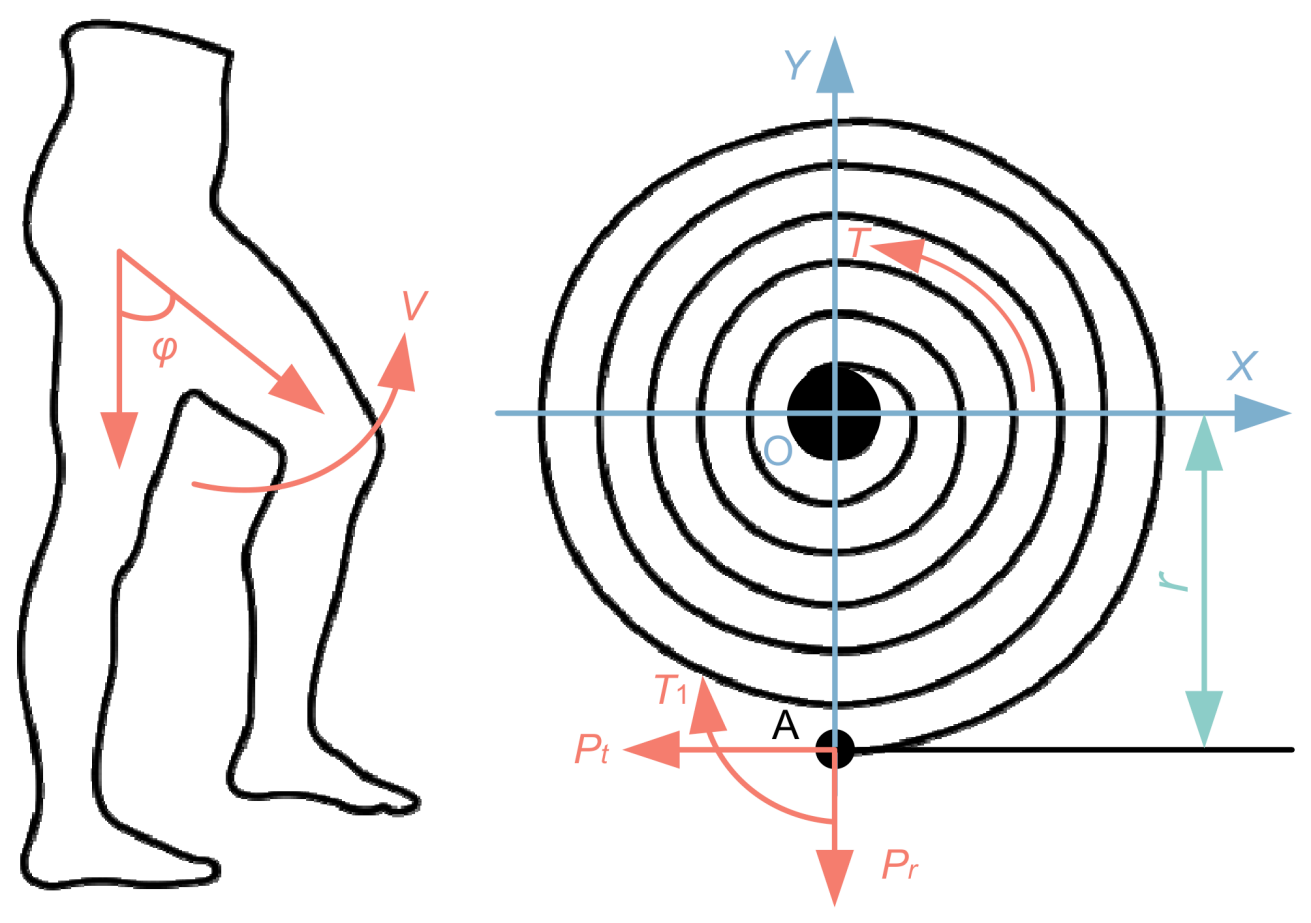
Force analysis of spiral spring.

The spiral spring is an important mechanical component commonly used in various mechanical and electronic devices, and its model is shown in Figure 3. When the free state of the spiral spring coincides with the reference point O, the working parts of the spring box and non-spring box are the same, including the spring plate, spring lock ring, etc. However, due to the different states of the spring, its elastic characteristics will change, so the idle speed when they reach the working parts is also different. In addition, factors such as the material and manufacturing process of spiral springs can also affect their elastic properties, which need to be selected and designed according to specific usage requirements.

When the spring is in working condition, the deformation angle φ and torque T of the spiral spring exhibit linear variation characteristics [15]. Therefore, the deformation angle φ of the spiral spring is directly proportional to the bearing torque T, that is

(2) $$\varphi =\frac{T}{K}$$

where K is the stiffness of the coil spring. The tail end of the spiral spring is fixed, and the fixed point is marked as point A. The distance between it and the center O of the spiral spring is represented as r. When torque T is applied to the shaft, point A will be affected by torque $T_{1}$, tangential force $P_{t}$, and radial force $P_{r}$, as shown in Figure 3.

In order to describe the infinitesimal displacement ds of the spring element along the spring length s, the variable spring deformation energy is introduced here, represented as dU. This value can be calculated by integrating the stress and displacement of the spring element. The deformation energy of a spring is related to its material properties, size, and stress distribution, and is an important parameter used to describe the elastic energy stored in the spring. The deformation energy dU of the spring in this unit is obtained

(3) $$dU=\frac{T^{2}ds}{2EI}$$

where E represents the elastic modulus of the material, I represents the moment of inertia of the material cross-section, and T represents the torque acting on that cross-section. When the effective length of the spiral spring is l, the deformation energy U of the spring can be obtained by integrating the entire length along the curve.

(4) $$U=\int_{0}^{l}dU=\int_{0}^{l}\frac{T^{2}}{2EI}d_{s}=\frac{T^{2}l}{2EI}$$

Equation (4) is an important parameter used to describe the storage of elastic energy in a spiral spring. This formula is based on the mechanical properties of materials and the geometric shape of springs, which can help to better understand the behavior and response characteristics of springs.

After the torque T is applied to the shaft, the spiral spring bears the same torque throughout its entire length, which is equal to the torque applied to the shaft. According to the card theorem in mechanics, the rate of change of strain energy U from an elastic member to a certain position P on the member is equal to the position δ corresponding to the load P, expressed by the formula δ=∂U/∂P. Therefore, the deformation angle φ of the spiral spring can be obtained as follows.

(5) $$\varphi =\frac{\partial U}{\partial T}=\frac{Tl}{EI}$$

The expression for the rotational stiffness K of the spiral spring obtained from Equations (2) and (5) is as follows:

(6) $$K=\frac{EI}{l}$$

In addition, another expression for the energy storage formula of the spiral spring obtained from Equations (4) and (5) is as follows:

(7) $$U=\frac{1}{2}T\varphi$$

This study chooses the elastic potential energy of the spiral spring, which is denoted as $E_{s}=U$. The deformation angle is represented by the number of deformation cycles n. Due to φ=2πn, the number of working coils of the spiral spring is as follows:

(8) $$n=\frac{Tl}{2\pi EI}$$

The size selection and performance calculation of spiral springs are based on the design and calculation of flat spiral springs in JB/T7366-1994 [16]. This study selected a 60Si2Mn spring steel strip with a thickness h of 1 mm and a width b of 190 mm as the material for the planar spiral spring of the device. Its elastic modulus E is 206 GPa, tensile strength $\sigma_{b}$ is 1863 MPa, density ρ is 7850 kg/$m^{3}$, and spring length l is 1800 mm.

(2) Cylindrical spiral spring selection

At present, there are three ways to achieve variable stiffness cylindrical spiral springs: variable pitch, variable pitch diameter, and variable wire diameter. However, compared with ordinary cylindrical spiral springs, variable stiffness cylindrical spiral springs have significant difficulties in process and design, especially when reverse engineering their structural parameters based on known spring stiffness characteristics. Therefore, this article adopts a discretization method to reverse-design the variable stiffness cylindrical spiral spring. Specifically, the spring is divided into *n* small segments, with each segment connected in series, and assuming that the stiffness of each segment is constant. During this compression process, the stiffness of the spring can be expressed as

(9) $$\left\{\begin{array}{c} \begin{array}{c} \frac{1}{K_{1}}=\frac{1}{k_{1}}+\frac{1}{k_{2}}+\cdots +\frac{1}{k_{n}}\\ \frac{1}{K_{2}}=\frac{1}{k_{2}}+\frac{1}{k_{3}}+\cdots +\frac{1}{k_{n}} \end{array}\\ \begin{array}{c} \cdots \cdots \cdots \\ \frac{1}{K_{n}}=\frac{1}{k_{n}} \end{array} \end{array}\right.$$

where $k_{1}$, $k_{2}$,…, $k_{n}$ are the stiffness of each small segment of the spring. From Equation (9), it can be inferred that

(10) $$\left\{\begin{array}{c} \begin{array}{c} k_{1}=\frac{K_{1}K_{2}}{K_{2}-K_{1}}\\ k_{2}=\frac{K_{2}K_{3}}{K_{3}-K_{2}} \end{array}\\ \begin{array}{c} \cdots \cdots \cdots \\ k_{n}=K_{n} \end{array} \end{array}\right.$$

(11) $$f=\frac{\left(F_{1}-F_{0}\right)}{K_{1}}+\frac{\left(F_{2}-F_{1}\right)}{K_{2}}+\cdots +\frac{\left(F_{n}-F_{n-1}\right)}{K_{n}}$$

Due to the known stiffness characteristics of the spring, the stiffness values of each small section of the spring can be obtained according to Equation (10), and the axial deformation when the spring is fully compressed can be calculated by Equation (11). When a variable stiffness cylindrical spiral spring is subjected to axial load, as the load increases, the spring will gradually merge and its stiffness will also gradually increase.

According to references [17,18], this article uses beam elements or solid elements to divide the mesh of springs. The advantage of beam elements is that they have fewer nodes and elements, and have a smaller computational scale. This article uses hexahedral elements with relatively fewer nodes and elements to partition the grid. Firstly, select one end face of the spring for mesh division, and then sweep the mesh along the spiral line to obtain a high-quality hexahedral mesh. The basic parameters of the spring are detailed in Table 1.

**Figure 4:**
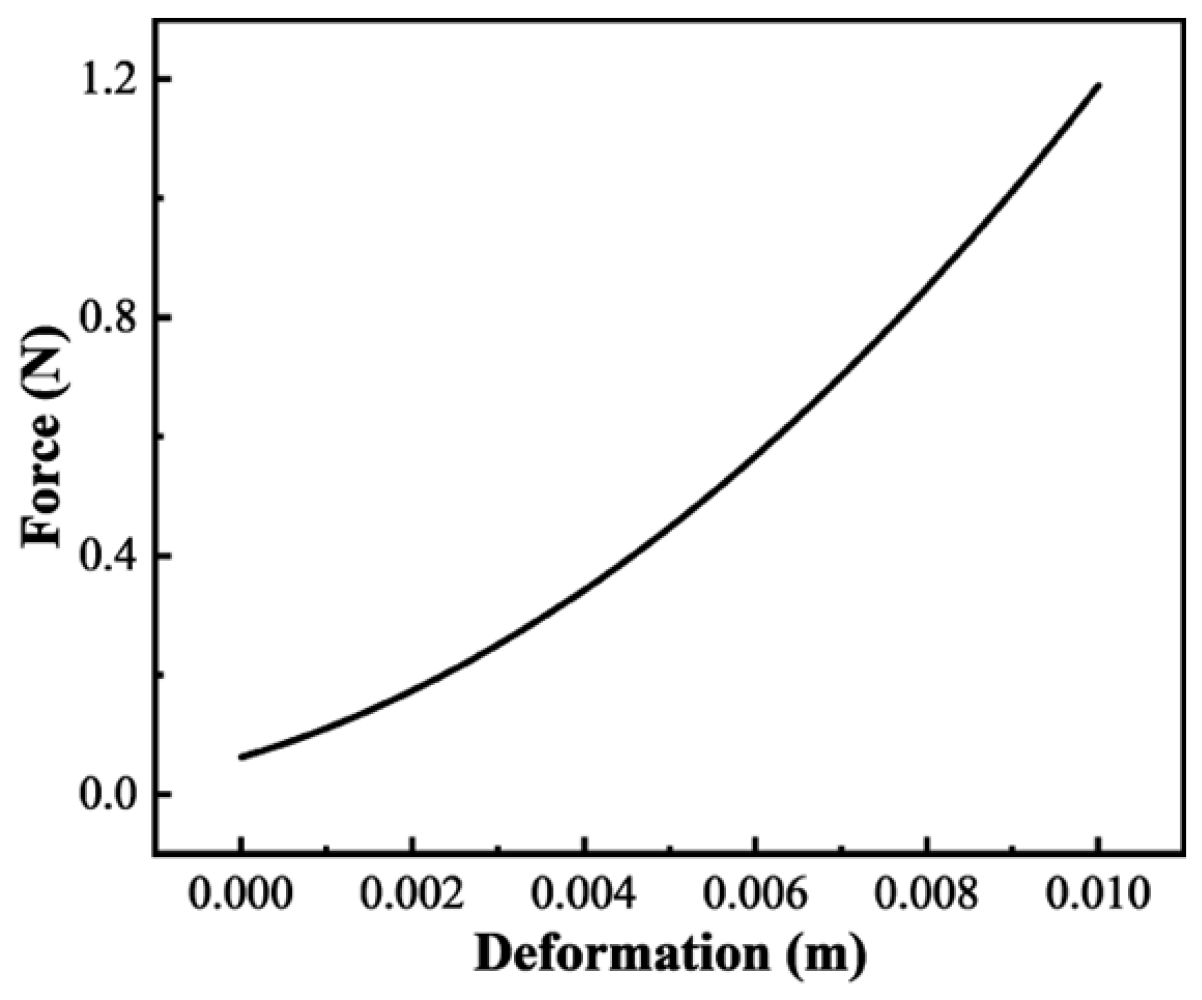
Load and deformation relationship curve of spring.

The numerical simulation process of the compression spring was carried out based on the commonly used LS-DYNA analysis module in ANSYS 2023 R2 finite element software. In terms of contact settings, consider the contact between the spring and the upper and lower spring seats and the contact between the spring itself. In terms of constraints, the two end faces of the spring were restrained, and an axial load was applied to the upper support of the spring. As shown in Figure 4, based on the finite element simulation results, the relationship curve between load and deformation can be obtained, which can quickly understand the stiffness characteristics of variable stiffness cylindrical spiral springs and compensate for some possible shortcomings in testing spring stiffness through experiments.

### 2.3. Hip-Assisted Exoskeleton Description

**Figure 5:**
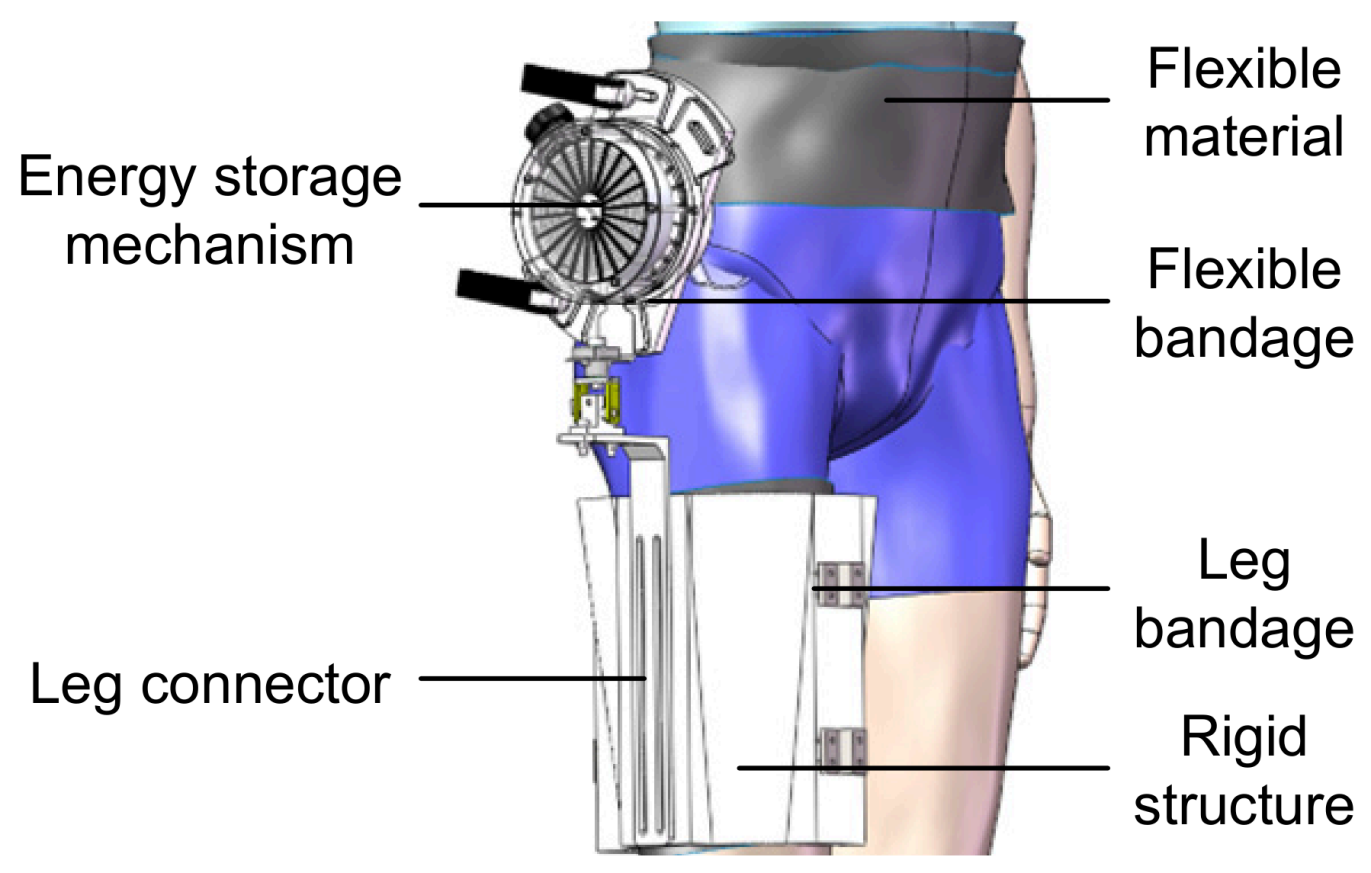
Appearance molding drawing of hip joint exoskeleton.

In the unpowered hip-assisted exoskeleton scheme, the man-machine binding interface is mainly set at the waist and thigh, and the form of combining rigid body and flexible body is adopted. The flexible material is in direct contact with the human body, and the rigid material is connected with the components of the exoskeleton device, as shown in Figure 5. The user can adjust the waist size according to the form of the waist strap to adapt to different body types. The binding structure adopts the form of a nylon fastener to ensure the convenience of the exoskeleton. The energy collection and conversion mechanism is fixed on the hard connecting plate. A soft pad is attached to one side of the hard connecting plate to connect with the belt, and a soft pad is also attached to the inside of the leg strap to provide comfort. The connecting part between the leg strap and the leg connecting rod is an adjustable rod with adjustable length to adapt to people of different heights.

**Figure 6:**
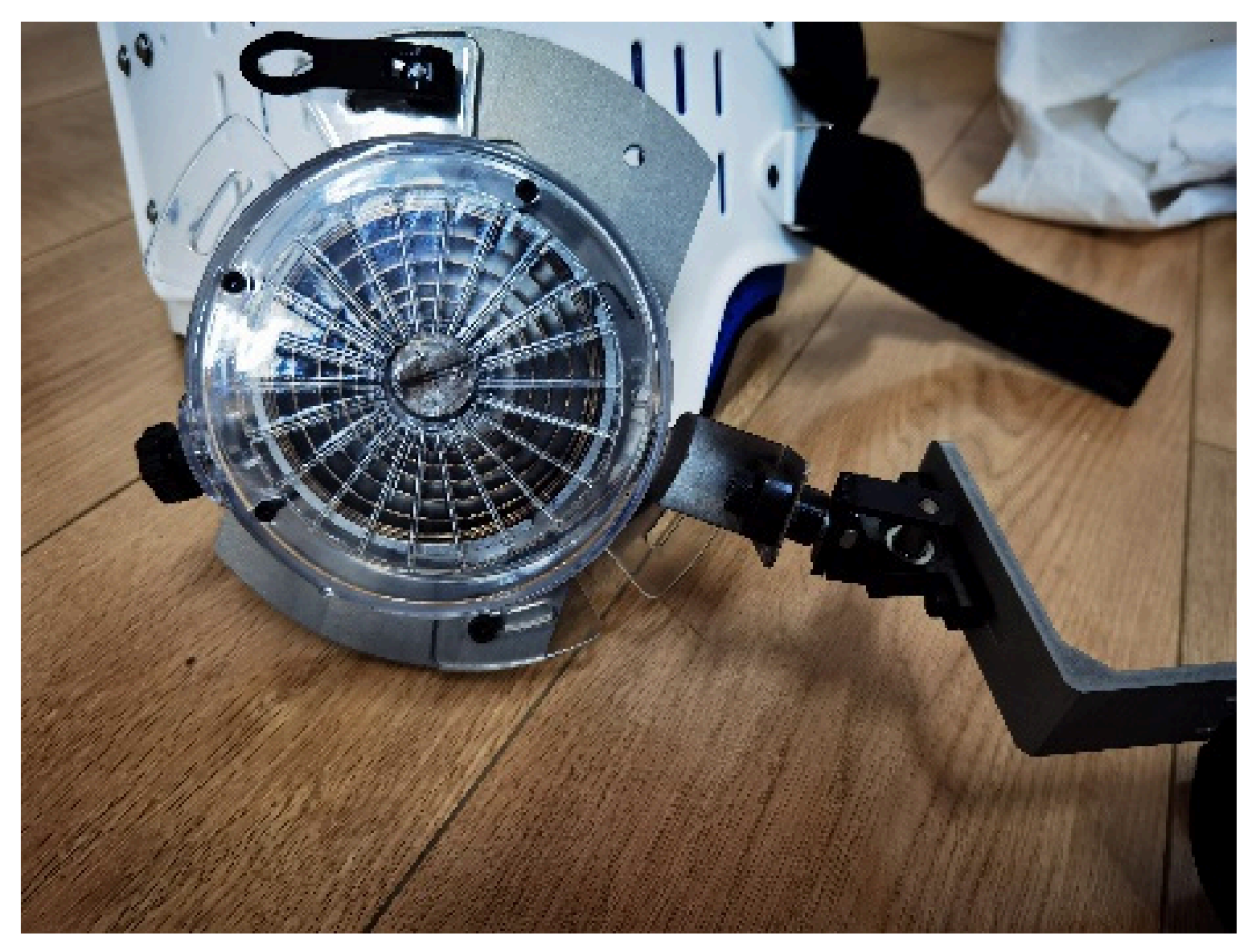
Physical image of hip joint prototype.

**Figure 7:**
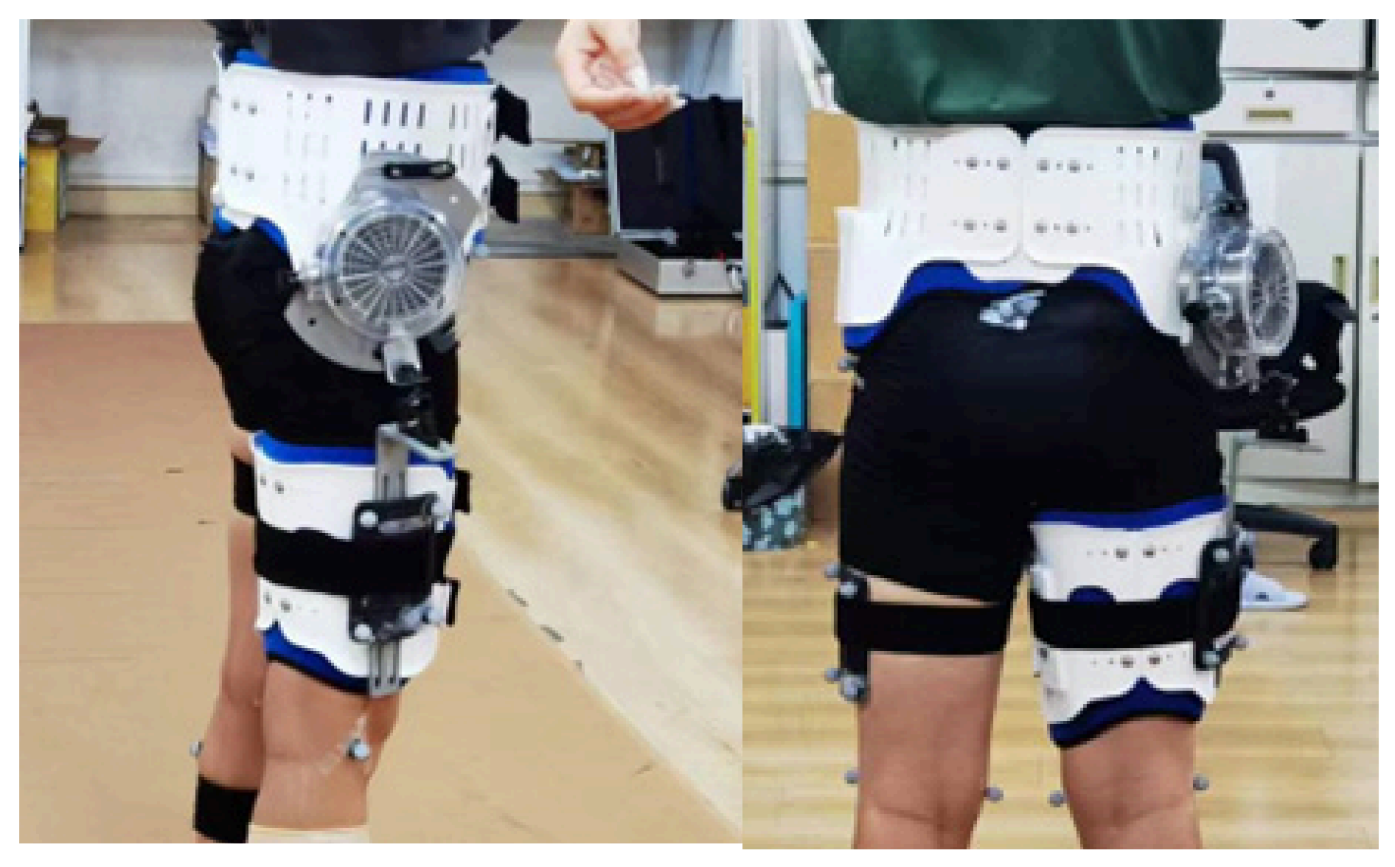
Wearing effect diagram of hip exoskeleton.

In order to verify the assistance effect and man-machine coupling of exoskeletons, it is necessary to design, process and assemble the internal parts of the exoskeleton. Through the processing and assembly of the physical model, we can verify whether it can work normally. The assembly effect of the mock-up is shown in Figure 6, and the wearing effect is shown in Figure 7.

**Figure 8:**
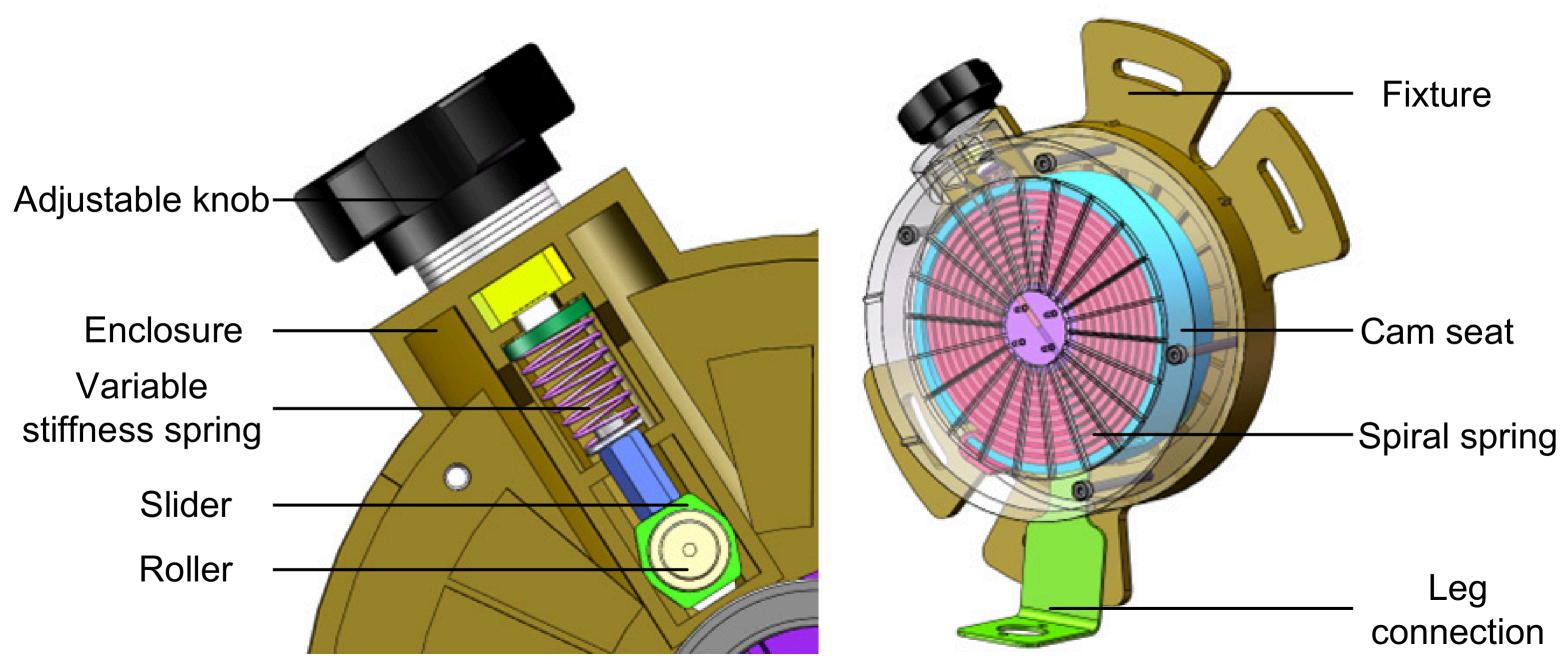
Internal structure of energy storage mechanism.

As shown in Figure 8, in actual use, the user wears the hip joint assisted exoskeleton at the waist position, and the two leg bandages are respectively fixed on the wearer’s left and right femurs. When the wearer wears the belt, the lower leg connecting rod is required to fit the user’s left and right tibias. When the user presses the leg rod, the leg rod drives the cam to rotate, and the coil spring installed on the cam shrinks, generating elastic potential energy. At the same time, the cam starts to push, acting on the grooved rolling bearing, and the bearing drives the groove to press the variable stiffness spring vertically downward, and the variable stiffness spring compresses, generating elastic potential energy.

In order to adapt to the walking of users with different forces, the unpowered hip joint assisted exoskeleton is equipped with a rotary adjustment knob for adjusting the preload of the variable stiffness spring above the pagoda deformation stiffness spring. The knob can adjust the preload of the spring by rotating clockwise and counterclockwise. Clockwise rotation increases the preload and counterclockwise rotation decreases the preload. The pre-tightening force adjusting screw is connected to the spring base, and above the spring base is a variable stiffness spring. When the preload adjusting screw is rotated clockwise, the spring base moves upward and the end coil under the variable stiffness spring is compressed, thereby increasing the preload. Conversely, when rotating counterclockwise, the preload will be reduced.

**Figure 9:**
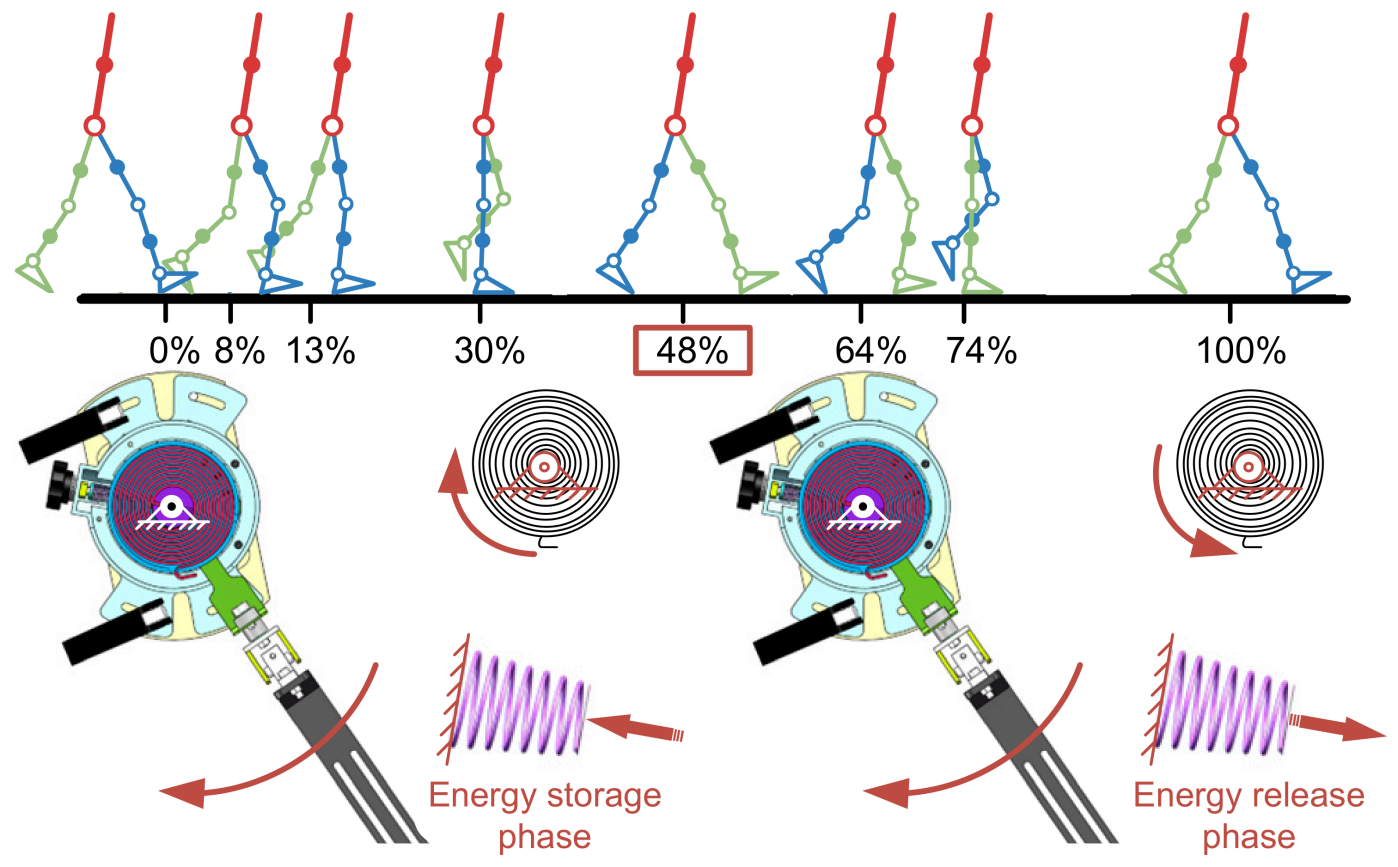
Hip exoskeleton energy storage and release.

The structural motion principle of the unpowered hip joint assisted exoskeleton is shown in Figure 9. First, the exoskeleton belt is fixed at the waist of the human body, so that the leg rod is suspended in the air and in close contact with the thigh. Fix it in the center of the thigh through the leg strap. When the lower limb of the human body is stretched, the rod of the leg is pulled to move backward, the cam rotates to drive the roller to slide, and the variable stiffness spring is compressed to store the initial mechanical energy. In the first 48% of the human walking support phase, the exoskeleton converts the initial mechanical energy into elastic potential energy and releases energy by assisting human flexion and leg lifting. At the later stage of the human walking support phase, the leg connecting rod is pressed down through the extension of the human leg, and the leg connecting rod is connected with the cam. The cam rotation compresses the deformation stiffness spring of the pagoda, and the scroll spring is stretched, which converts the mechanical energy of human walking into elastic potential energy to complete the collection of energy. At the early stage of the swing phase of human walking, the human leg flexes, and the cam rotates with the movement of the human leg, so that the deformation stiffness spring of the pagoda rebounds, and the coil spring retracts, releasing the elastic potential energy to assist the human leg lifting.

To quantitatively evaluate the assistive power delivered by the exoskeleton, the deformation of the coil spring and the variable stiffness spring was derived from the rotation angle of the cam, which was recorded synchronously with the motion capture system. Based on the pre-calibrated stiffness coefficients of the springs, the elastic forces were calculated from the measured deformations. The instantaneous assistive torque at the hip joint was then obtained through the transmission relationship between the cam and the leg linkage, as illustrated in the kinematic diagram of Figure 9. The instantaneous assistive power was computed as the product of the assistive torque and the hip joint angular velocity, which was derived from the motion capture data. This method enables a direct evaluation of the power provided by the exoskeleton throughout the gait cycle, complementing the indirect assessment via metabolic energy reduction and changes in joint power.

The unpowered hip joint-assisted exoskeleton plays a role in the changes of stretching and flexion during the gait cycle of the user through the periodic assistance effect. It can timely obtain the mechanical energy generated by the human body through the energy collection and conversion structure, and convert it into elastic potential energy, so as to realize the real-time collection and release of energy and achieve the purpose of real-time assistance. The pagoda deformation stiffness of the unpowered hip exoskeleton is also equipped with a rotary handle with adjustable preload under the spring, which can adjust the preload of different springs to adapt to wearers with different forces, so as to achieve better human-computer coordination performance.

## 3. Methods

### 3.1. Construction of CE-ULLWE Method

**Figure 10:**
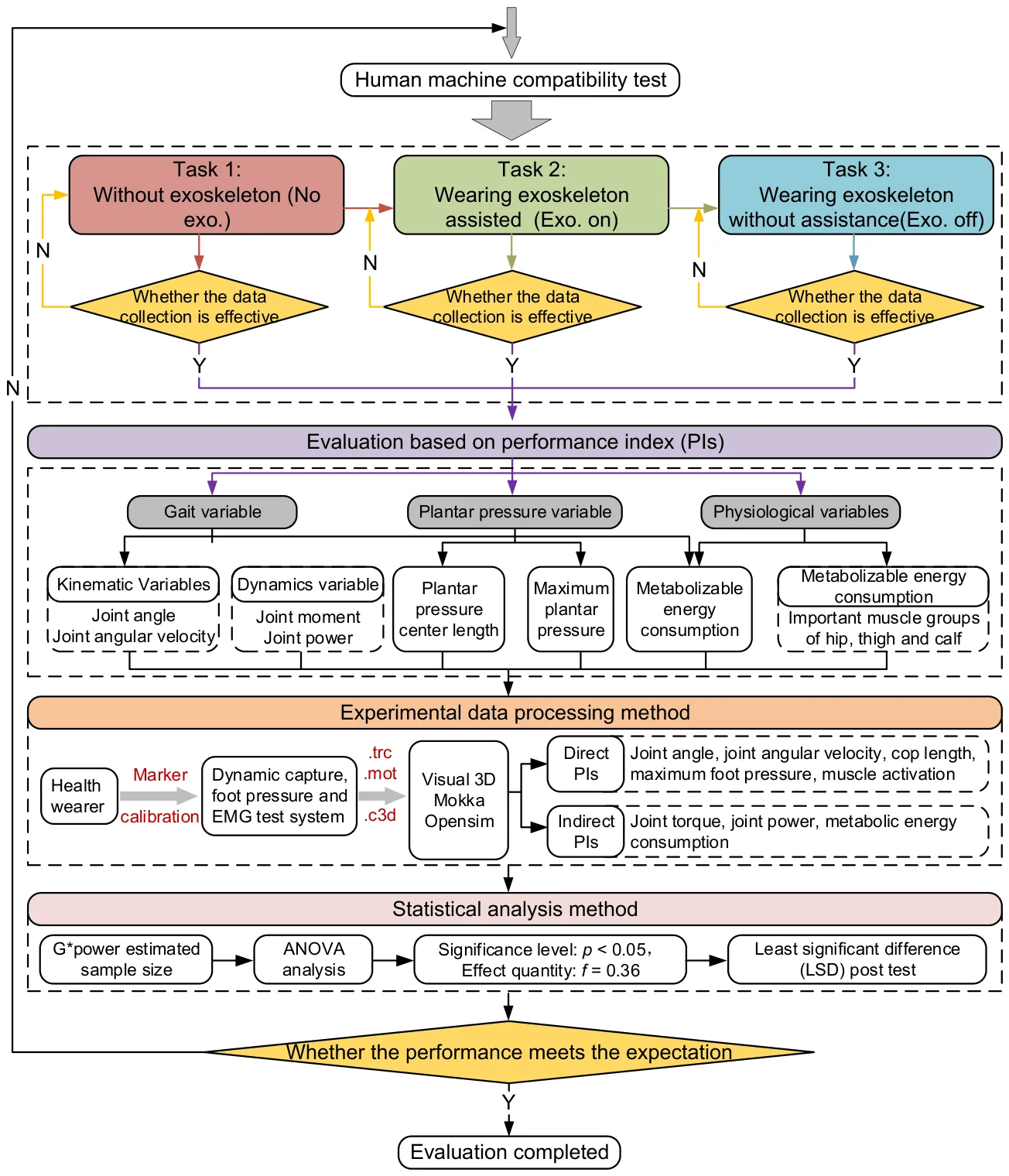
CE-ULLWE model flowchart.

The unpowered lower limb wearable booster exoskeleton is used to reduce human metabolic energy consumption, delay fatigue and increase body speed, etc. It focuses on healthy people who can perform certain tasks without the exoskeleton. The current wearable exoskeleton evaluation system is multi-faceted and dynamic, with a focus on driving validation and algorithm performance, while lacking targeted standardization protocols for passive lower limb exoskeletons [19]. The existing indicators exhibit a discrete distribution and are mostly limited to a single dimension such as metabolic energy consumption or joint torque, making it difficult to integrate human–machine compatibility, multiple working conditions, and multi-source biomechanical information to comprehensively characterize the assistance effect [20]. Therefore, the CE-ULLWE model proposed in this study is associated with a series of basic PIs for non-dynamic assistance of lower limb exoskeleton, as shown in Figure 10. The comprehensive evaluation model specifically includes the following Phases.

Phase 1: Human machine compatibility test. The subjects need to understand and be familiar with the basic functions and wearing precautions of the tested exoskeleton device. Before the formal measurement task, the subjects were familiar with the tested exoskeleton through the wearing test and achieved the best human-computer compatibility.

Phase 2: Formulate phased experimental tasks. In order to evaluate the function of the exoskeleton, the performance test of the exoskeleton in three cases was proposed, including “No exo.”, “Exo.on” and “Exo. off”. The “Exo. off” is tested to evaluate the impact of the quality of the wearable exoskeleton on its performance, which is an important consideration in the design of a wearable exoskeleton system.

Phase 3: Evaluate based on performance indicators. In the current work, the basic performance indicators are clustered into gait (kinematics and dynamics) variables, plantar pressure variables and physiological variables to comprehensively quantify the performance effectiveness of the tested exoskeleton.

(1) Gait variables. In gait variables, kinematics and dynamics variables are included. The joint angle and joint angular acceleration in the kinematic variables are considered to be the most important indicators to characterize the motion state. The change of rotation angle in the whole test process can be determined by the joint angle variable, and the dynamic auxiliary feedback and motion attitude can be directly characterized by the joint angular acceleration variable. However, the dynamic variables cannot be measured directly by the experimental device, and the ground reaction force must be used as the input value for simulation, which will lead to inaccuracy relative to the kinematic variables. It is usually not used to represent the main body movement, but it can still be used as one of the gait evaluation indicators.
(2) Plantar pressure variables. In the process of foot movement, the center of pressure (COP) and the maximum plantar pressure in plantar pressure variables are important biomechanical information to characterize limb movement. It is closely related to different gait movements, which correspond to different pressure signal generation characteristics [21].
(3) Physiological variables. The most commonly used physiological variables are metabolic consumption and muscle activity, which are often used to evaluate the body’s exercise status and fatigue. Among them, metabolic consumption includes oxygen intake, carbon dioxide output, respiratory rate, heart rate and other indicators, which can reflect the energy and coordination mechanism required by the body during exercise. Muscle activity is evaluated to evaluate the degree of muscle contraction and relaxation through EMG signals, so as to understand muscle fatigue and exercise performance.

Phase 4: Experimental data processing method. Vicon Nexus (Vicon Nexus 2.6.7), Mokka, Visual 3D (C-Motion Inc., Germantown, MD, USA), OpenSim (Version 4.1), Qualisys Track Manager (Version 2018), BioWare and other software are used to realize the pretreatment of experimental data, format conversion, single cycle selection, kinematics and dynamics analysis.

Phase 5: Statistical analysis method. The sample size was estimated using G*Power 3.1.9.7, and the one-way repeated measurement analysis of variance was used as the statistical method. The significance level was set to *p* = 0.05, and the effect quantity was f = 0.36. In order to achieve 80% statistical efficacy, the minimum sample size is 14. In order to further improve the statistical efficacy, each group of tasks in the test experiment will ensure the collection of 15 groups of effective data.

Early exoskeleton evaluation research mainly focused on verifying control performance, using dispersed technical indicators for device-level validation. The common evaluation methods can be classified into three paradigms, such as biomechanical indicators, physiological metabolic evaluation, and human-computer interaction and usability evaluation [22]. However, each method is independent of the others and lacks systematic integration. Pinto Fernandez et al. (2020) further pointed out in their systematic review that the evaluation of lower limb exoskeletons is still highly focused on straight walking scenarios, with a serious lack of coverage of basic motor skills in daily life, and there is a clear bias towards general kinematic and dynamic indicators [23].

Therefore, the CE-ULLWE model achieved breakthroughs at three levels. Firstly, the experimental design introduces the “Exo. off” independent working condition, achieving a quantitative separation of the impact of the exoskeleton’s own quality and the contribution of assistance efficiency, breaking through the limitations of traditional dual working conditions (with/without assistance) that cannot identify structural negative effects and auxiliary positive effects, providing a new approach for quality efficiency decoupling evaluation. Secondly, the evaluation indicators break through the limitations of a single type of parameter, clustering the basic indicator system into three categories: gait variables (covering kinematics and dynamics), plantar pressure variables, and physiological variables. Through multi-source information fusion, the comprehensive impact of exoskeletons on the wearer’s movement patterns, ground interaction, and metabolic load is synchronously evaluated, overcoming the problem of insufficient representation ability of single-dimensional indicators. Thirdly, the evaluation process includes human–machine compatibility testing as a prerequisite, requiring the wearer to reach the optimal adaptation state before conducting formal measurements, shifting the evaluation perspective from “device performance verification” to “overall human–machine system efficiency”, ensuring that the results are more closely aligned with practical application scenarios. In summary, the CE-ULLWE model provides a systematic, scientific, and reproducible comprehensive evaluation framework for unpowered lower limb exoskeletons through three innovative approaches, including three-condition comparative design, multi-source indicator clustering system, and compatibility pre-evaluation.

### 3.2. Participants

The participants involved in the performance evaluation of the hip-assisted exoskeleton consisted of one healthy adult female (age: 23 years old, height: 168 cm, weight: 49 kg) and one healthy adult male (age: 25 years old, height: 175 cm, weight: 62 kg). Neither participant had a history of musculoskeletal disorders, surgical interventions, or any other relevant medical conditions. Prior to the commencement of data collection, the objectives and procedures of the experiment were thoroughly explained to the participants. Gait measurements were conducted only after informed consent had been obtained in accordance with the prescribed experimental protocol.

The passive lower-limb wearable exoskeleton developed in this study incorporates elastic components arranged in parallel with the human lower extremity during the stance phase. Nevertheless, the overall mass of the device and its attachment configuration may influence the wearer’s natural movement characteristics. The additional weight imposed by the exoskeleton generates gravitational loading on the hip extensor and knee flexor muscle groups, as these muscles are required to elevate the device during the initial swing phase. Furthermore, the increased mass contributes to greater inertial effects on the hip flexor muscles because additional acceleration is needed to propel the limb-exoskeleton system while walking. Since the incorporation of external mass inevitably increases inertial forces during locomotion, and complete body attachment without imposing movement constraints is difficult to achieve, reductions in joint mobility and user discomfort may occur.

To determine whether the exoskeleton can effectively reduce metabolic expenditure during load-bearing walking, experimental assessments were carried out under three distinct conditions: “No exo.”, “Exo. on”, and “Exo. off”. The “Exo. off” condition was specifically designed to isolate and quantify the influence of the exoskeleton’s own mass on walking performance, which represents a critical factor in wearable exoskeleton design and optimization. Throughout all testing sessions, the device was mounted exclusively on the participant’s right lower limb.

### 3.3. Instrumentation and Data Collection

**Figure 11:**
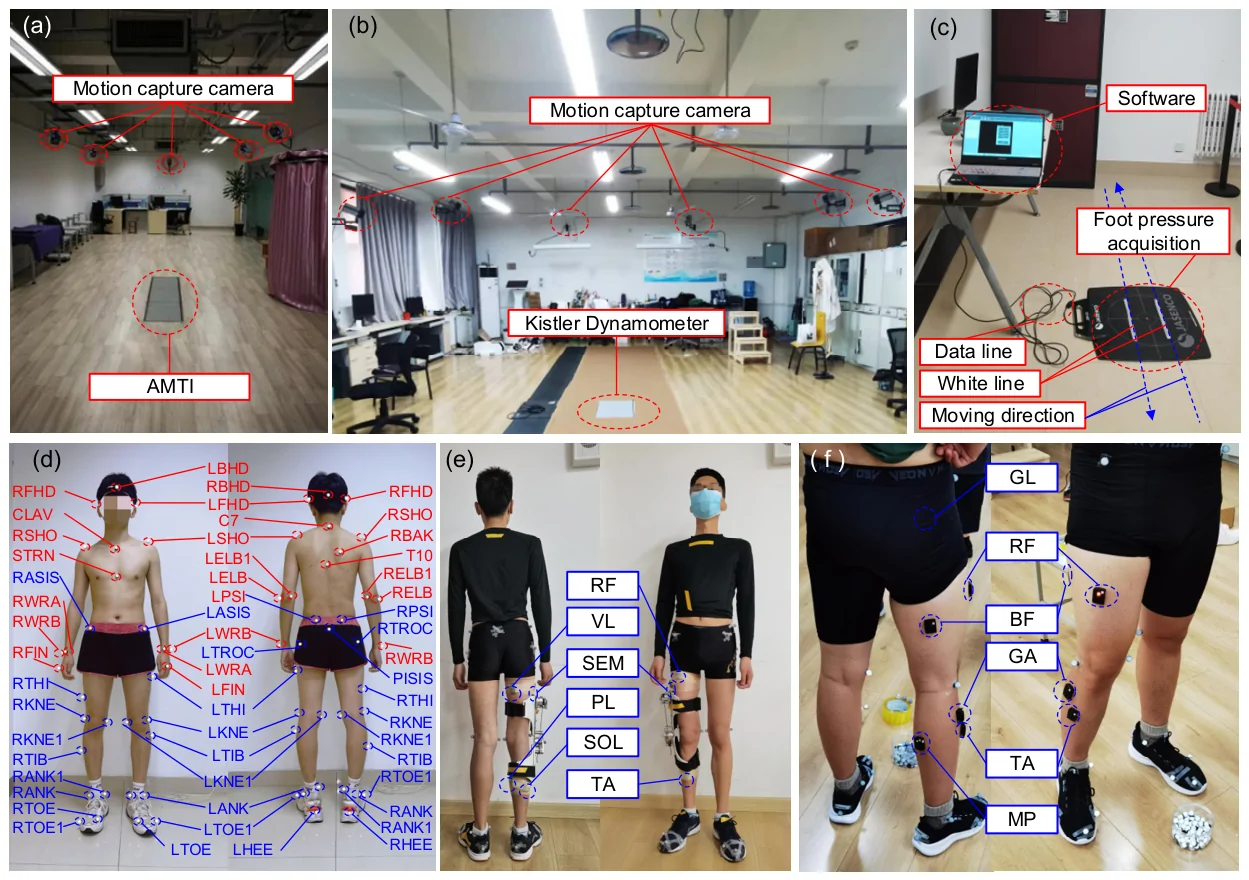
(**a**) Vicon motion capture experiment; (**b**) Qualisys motion capture system; (**c**) Distributed plantar pressure experiment; (**d**) Body marker points; (**e**) Knee and (**f**) hip exoskeleton electromyography testing.

(1) The three-dimensional motion measurement and analysis platform employed in this study primarily consisted of the following components. A three-dimensional infrared motion capture system manufactured by Vicon Motion Systems (VICON T40S, Vicon, Oxford, UK) was utilized in conjunction with the Vicon optical motion analysis platform. The experimental setup included multiple motion capture cameras, three AMTI force plates, a data acquisition computer, reflective markers, a calibration frame, and other supporting accessories, as illustrated in Figure 11a. The sampling frequency of the motion capture cameras was configured at 100 Hz, whereas ground reaction force data from the force platforms were recorded at 1500 Hz. During testing, reflective markers were attached to predefined anatomical locations on the participant’s body. These markers reflected infrared light of a specific wavelength, enabling the cameras to continuously identify and track their spatial positions. Subsequently, kinematic and kinetic data required for gait analysis were processed and collected using the Vicon Nexus motion analysis software package.

Qualisys motion capture system is adopted with a shooting frequency of 200 Hz, and a Kistler three-dimensional dynamometer (9287C, 900 × 600 × 200 mm, Switzerland) is used with a sampling frequency of 2000 Hz, as shown in Figure 11b. Its motion analysis software uses Qualisys Track Manager (Version 2018).

NOKOV three-dimensional optical motion capture system (NOKOV Science & Technology Co. Ltd., Beijing, China) was employed to obtain kinematic information during the experiments. The measurement platform mainly consisted of eight optical cameras, a central control workstation, and a set of reflective markers attached to the subject. Owing to the retroreflective coating on the marker surfaces, incident light emitted by the cameras could be effectively reflected and subsequently recognized by the system. By tracking the spatial coordinates of these markers in real time, the platform was capable of acquiring motion-related parameters, including displacement, velocity, and acceleration.

(2) The EMG test system uses the American Delsys (Trigno Wireless EMG System) wireless surface muscle signal flow measurement system to collect the EMG signals of the lower limbs. The system can collect 8 channels of EMG signals synchronously and wirelessly. The data transmission range of the wireless sensor can reach 40 m. The size of the sensor is 37 × 2 × 15 mm, the weight is less than 15 g, and the sampling frequency is 2000 Hz.
(3) Plantar pressure data were collected using a distributed plantar pressure measurement system (JASENCO, JSP-C5, France). As illustrated in Figure 11c, the testing apparatus primarily comprised pressure sensing and acquisition units, signal transmission cables, a computer for data processing, and dedicated software for data collection and analysis.

### 3.4. Experimental Procedure

During the experiment, the kinematics and dynamics of the joint were measured. The improved Cleveland Clinic pelvic and leg marker sets (left and right ASIS, left and right greater trochanter, left and right PSI, pubic marker points, thigh marker points, medial epicondyle and lateral epicondyle, calf marker points, medial and lateral malleolus, calcaneus, foot, fifth metatarsal) were used for the lower limbs of the experimenters. Two groups of pasting methods were used during the experiment: one was whole-body pasting, and the other was only applied to the lower limbs, as shown in Figure 11d. Following the completion of static standing calibration, the joint kinematic parameters, kinetic characteristics, and center-of-mass trajectories were reconstructed using Visual 3D software. During the modeling procedure, bilateral lower limbs were assumed to exhibit symmetrical biomechanical properties.

Before the formal data collection, all participants received a comprehensive introduction to the experimental protocol and underwent at least 30 min of equipment training every day. Specifically, a structured familiarization session was conducted on the day prior to the formal experiments, which included static fitting and adjustment (5 min), slow-speed walking adaptation (10 min) on level ground, and self-paced walking practice (15 min) on a treadmill with a safety fall-arrest harness. The total familiarization duration per session was thus 30 min, and this same duration was applied to all participants. A 5-min warm-up was performed on each testing day before data collection, and the familiarization session was separated from formal data collection by at least 24 h to minimize learning and fatigue effects. Gait parameters (e.g., step length and cadence) were monitored during familiarization, and stabilization was defined as a coefficient of variation of less than 5% across three consecutive walking trials; only after this criterion was met did participants proceed to formal testing. Adaptation training was initially conducted on level ground, after which participants practiced on a treadmill while wearing a safety fall-arrest harness. To ensure operational safety throughout the training process, subjects were instructed to maintain a sufficiently wide step width to avoid interference between support structures. In addition, adequate knee extension was required so that the locking mechanism could function effectively during standing.

To control for order effects and potential learning across repeated trials, the sequence of the three experimental conditions (“No exo.”, “Exo. on”, “Exo. off”) was counterbalanced across participants using a block randomization scheme generated by a computer-based random number generator (www.randomizer.org) with balanced block sizes to ensure approximately equal numbers of participants assigned to each condition order. The experimenter who performed data collection was aware of the condition sequence, but subsequent data processing and statistical analysis were conducted blinded to condition assignment. A minimum rest period of 10 min was enforced between different conditions to eliminate residual fatigue.

**Figure 12:**
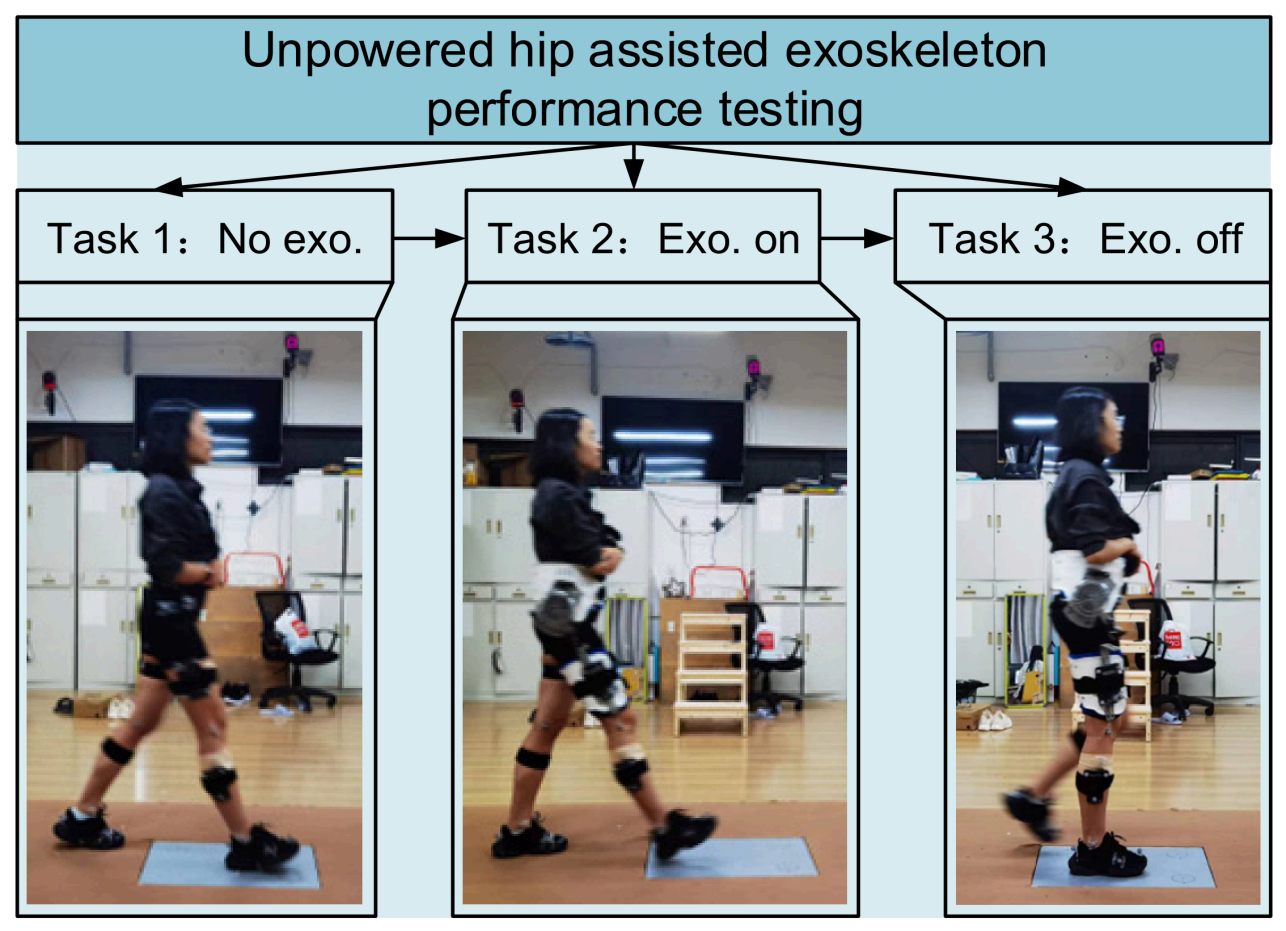
Unpowered hip-assisted exoskeleton performance testing scenario.

For the three-dimensional motion capture experiment (Figure 12), participants wore tight-fitting shorts and remained shirtless to minimize the possibility of reflective markers being obscured by clothing, thereby enhancing measurement accuracy. Before dynamic gait trials commenced, static calibration data were collected. During this procedure, subjects stood on a three-dimensional force platform with their feet positioned apart and their arms extended naturally. Subsequently, walking trials were performed. Participants were instructed to traverse a predetermined straight path approximately 5 m in length at a self-selected pace, ensuring that each foot sequentially contacted the three force plates embedded along the walkway. To maintain consistency across trials, walking cadence was regulated using a metronome, allowing participants to synchronize their steps with a uniform rhythm. One complete gait cycle was defined as walking forward across the force-measurement system and then returning to the starting position for the subsequent trial. Gait phase identification was accomplished through analysis of ground reaction force signals. For every walking condition, a total of 50 gait cycles were recorded. Furthermore, a 2-min recovery period was provided between successive trials to minimize the influence of fatigue on kinematic and kinetic measurements and to preserve the participants’ natural walking patterns as much as possible.

During the dynamic foot pressure test (Figure 12), the experimenter is required to walk through the plantar pressure measuring plate with his left foot, and then walk back with his right foot, so as to obtain the dynamic plantar pressure distribution of both feet. The experimental procedure was conducted according to the following protocol. Initially, participants positioned the device at the center of the testing area and walked along a straight 10 m pathway at a self-selected comfortable speed. During traversal of the pressure-sensing platform, subjects were instructed to align the tip of the foot with a predefined white reference line to ensure consistency among trials. To reduce the potential influence of fatigue on gait performance, five valid trials were recorded for each dynamic condition, and a resting period of 15 min was provided between consecutive tests.

During the wearable exoskeleton evaluation, muscle activation characteristics were employed as an important indicator for assessing assistive performance. A wireless surface electromyography (sEMG) acquisition system was utilized to monitor muscle activity, including muscle function, anatomical location, and activation depth. To investigate the effectiveness of the knee-assist exoskeleton (Figure 11e), six representative muscles were selected for analysis: rectus femoris (RF), vastus lateralis (VL), semitendinosus (SEM), tibialis anterior (TA), peroneus longus (PL), and soleus (SOL). For the hip-assist exoskeleton experiments (Figure 11f), the monitored muscles included rectus femoris (RF), biceps femoris (BF), gluteus maximus (GL), tibialis anterior (TA), gastrocnemius (GA), and medial peroneal muscle (MP). Comparative analysis of these muscles provided a quantitative basis for evaluating the assistance offered by the exoskeleton systems.

### 3.5. Data Processing

Owing to technical limitations associated with motion capture experiments, including marker occlusion, displacement of reflective markers, and the generation of spurious markers caused by reflections from the exoskeleton structure, a portion of the collected recordings could not be used for subsequent analysis. Consequently, the number of valid gait cycles differed among participants, making it impractical to analyze all 50 collected cycles for every trial. After preprocessing the complete dataset, 15 sets of high-quality and successfully reconstructed gait records were retained for detailed analysis. To further minimize the influence of fatigue, data segments obtained after at least one minute of continuous walking were preferentially selected, and the earliest valid reconstruction within this period was used for subsequent calculations.

Motion data acquisition was performed using the Vicon three-dimensional motion capture system, which generated gait files in .c3d format. The recorded marker trajectories were first examined and screened using Mokka software to identify complete and reliable gait datasets. Subsequently, the selected files were imported into Visual 3D for further biomechanical analysis. This process enabled extraction of plantar pressure information and identification of gait cycles suitable for investigation. In the present study, a complete gait cycle was defined as the interval between the first contact of the right foot and the subsequent right heel strike. Based on this definition, variations in knee joint angle, joint force, and joint torque throughout the gait cycle were calculated. Furthermore, pressure distribution data obtained from the force platform during the stance phase were extracted, allowing the generation and analysis of plantar pressure variation curves across the entire gait cycle. The instantaneous assistive power of the exoskeleton was calculated by multiplying the assistive torque (derived from spring deformation and cam transmission geometry) by the hip joint angular velocity obtained from the motion capture system. The integral of the positive assistive power over the gait cycle was used to quantify the total energy delivered by the exoskeleton.

**Figure 13:**
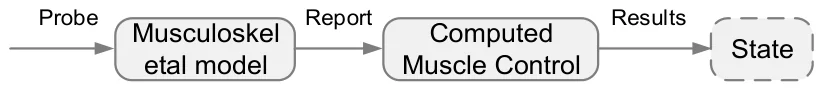
Metabolic probes in OpenSim.

The simulation analysis of human biomechanics and motion mode using OpenSim can be conducted by establishing personalized models [24,25] or according to the existing models [26] provided on the official website. The experimental data include position data (.trc), force data (.Mot) and electromyography (sEMG) data. With the support of the above experimental data, OpenSim can conduct simulation analysis in two ways: reverse motion analysis and forward motion analysis, and obtain the energy consumption rate of each muscle and the whole body with the help of the metabolic calculator in the software. The probe is used in conjunction with the forward tool and analysis tool, as shown in Figure 13.

The metabolic model proposed by Umberger et al. [27,28] explained the effects of muscle mass, the proportion of fast and slow muscle fibers in muscle, and the lengthening/shortening speed. Uchida et al. [29] continuously expanded the metabolic probe to calculate the energy consumed by all muscles and each muscle in the model. In the analysis of metabolic energy consumption, OpenSim can calculate metabolic rate and energy consumption by integrating the musculoskeletal system model, physiological model and mechanical model. By simulating muscle activities in different postures, OpenSim can quantitatively calculate the metabolic energy required by the human body when doing a certain action, and can find energy-saving strategies to provide scientific guidance for sports training, auxiliary assistance and rehabilitation treatment. The calculation equation of energy consumption in OpenSim is as follows,

(12) $$\dot{E}={\dot{h}}_{A}+{\dot{h}}_{M}+{\dot{h}}_{SL}+{\dot{w}}_{CE}$$

where, $\dot{E}$ is the total energy consumption, and the unit is usually J/s or W, ${\dot{h}}_{A}\left(u\left(t\right),a\left(t\right),r_{ST},S\right)$ is the activation heat generated by calcium ion transport, ${\dot{h}}_{M}\left(u\left(t\right),a\left(t\right),r_{ST},S\right)$ is the maintenance heat generated by actin interaction, ${\dot{h}}_{SL}\left(u\left(t\right),a\left(t\right),r_{ST},S,v_{CE}(t)\right)$ is the shortened/extended heat consumption rate calculated for the separation of fast and slow twitching fibers, ${\dot{w}}_{CE}$ is the mechanical power of the retractable element.

The electromyography (EMG) data were processed using MATLAB (Version R2021b, MathWorks, Inc., Natick, MA, USA). Raw EMG signals were filtered, rectified, and normalized according to standard procedures. Metabolic data were obtained through musculoskeletal modeling and simulation using the AnyBody Modeling System (Version 7.4, AnyBody Technology, Aalborg, Denmark), which allowed for the estimation of muscle metabolic energy expenditure based on the simulated muscle activations and joint kinetics.

### 3.6. Statistical Analysis

All statistical analyses were conducted using SPSS software (Version 26.0). Statistical significance was determined using a threshold of *p* < 0.05 for all tests. To evaluate the influence of the exoskeleton on gait characteristics and plantar pressure distribution, a one-way repeated-measures analysis of variance (ANOVA) was performed across three experimental conditions: “No exo.”, “Exo. on”, and “Exo. off”. In addition, differences in mean muscle activation over an entire gait cycle were examined using repeated-measures ANOVA under two conditions, namely “No exo.” and “Exo. on”, to assess the assistive effect of the device on neuromuscular performance.

G*power 3.1.9.7 was used to estimate the planned sample size (*α* = 0.05, 1 − *β* = 0.80). Considering the differences in research variables and the stability of results, if the interaction is to be observed in the experimental design of repeated measurement of two factors, all experiments were calculated based on the standard of a medium effect size (*f* = 0.25), and the result was 15 samples. Following the ANOVA procedures, pairwise comparisons were conducted using the least significant difference (LSD) post hoc test. This approach was adopted to identify the minimum statistically significant differences among experimental conditions when a significant main effect was detected. All inferential statistical evaluations were carried out according to the above analytical framework.

## 4. Results and Discussion

### 4.1. Gait Results and Analysis

**Figure 14:**
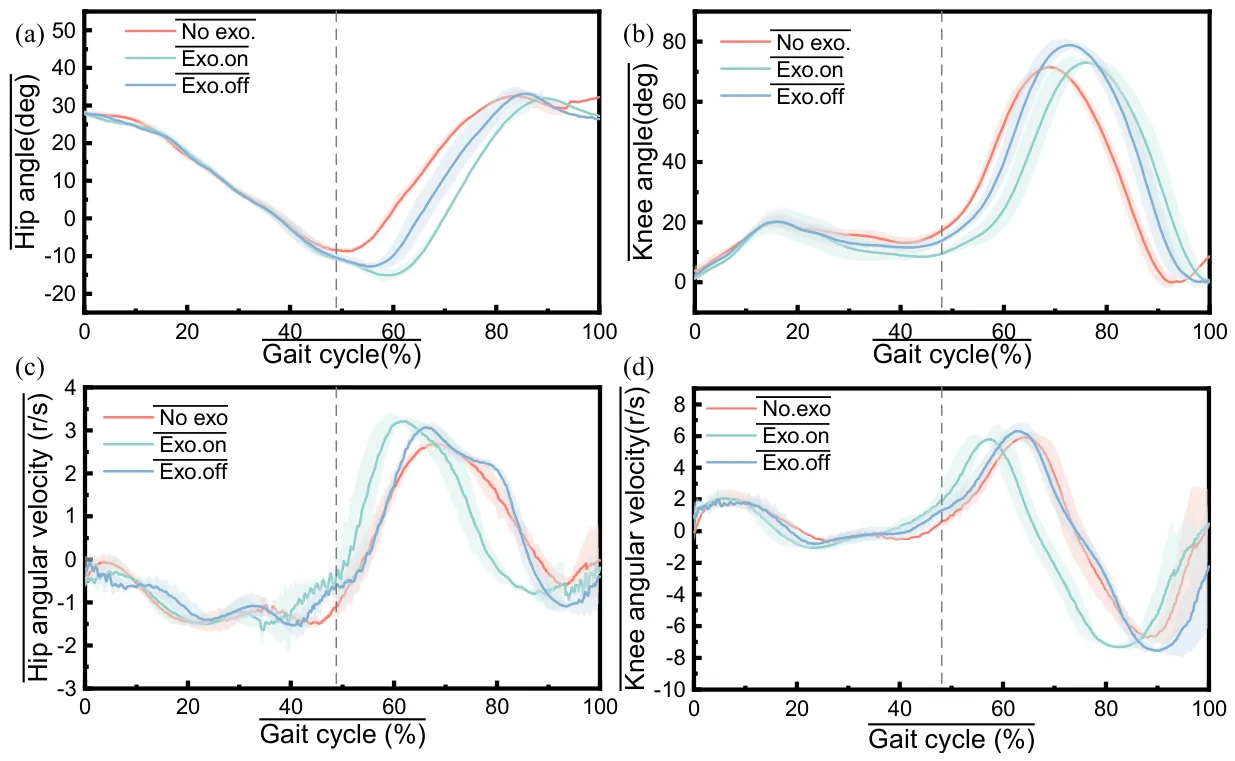
The effect of an unpowered hip exoskeleton on hip (**a**) and knee (**b**) joint angles and hip (**c**) and knee (**d**) joint angular velocity.

When the subjects were walking at their own speed, gait data were obtained under “No exo.”, “Exo. on” and “Exo. off” conditions. The changes in hip and knee joint angle and angular velocity when wearing an unpowered hip exoskeleton are shown in Figure 14. It can be seen from Figure 14a,b that in the “No exo.” state, the range of flexion and extension in the sagittal plane of the hip and knee joint is about 41.2° and 71.5°, respectively. In the “Exo. on” state, the range of flexion and extension in the sagittal plane of the hip and knee joint was about 47.1° and 71.7°, respectively, which increased by 14.3% (*n* = 39 groups; one-way repeated-measures ANOVA; F = 8.355, LSD post hoc analysis: *p* = 0.001) and 0.3% (LSD post hoc analysis: *p* = 0.940) compared with the “No exo.” state. It can be seen from 12C and D that the maximum angular velocity of the hip and knee joints in the “No exo.” state is about 2.7 R/s and −6.7 R/s, respectively. In the “Exo. on” state, the maximum angular velocity of the hip and knee joints was about 3.2 R/s and −7.3 R/s, respectively, which increased by 18.5% (LSD post hoc analysis: *p* < 0.001) and 9.0% (LSD post hoc analysis: *p* < 0.001) compared with the “No exo.” state. The performance test results of the unpowered hip-assisted exoskeleton showed that the hip-assisted exoskeleton had little effect on the angle and angular velocity of the hip and knee joints during the energy storage phase. When wearing a hip-assisted exoskeleton, the angle of the hip and knee joints increases significantly in the energy release stage, and the important thing is that the angular velocity of the hip joint increases significantly, which indicates that wearing the hip-assisted exoskeleton has the performance of enhancing the speed and range of motion of the human body, and will not limit the normal activities of lower limb joints.

**Figure 15:**
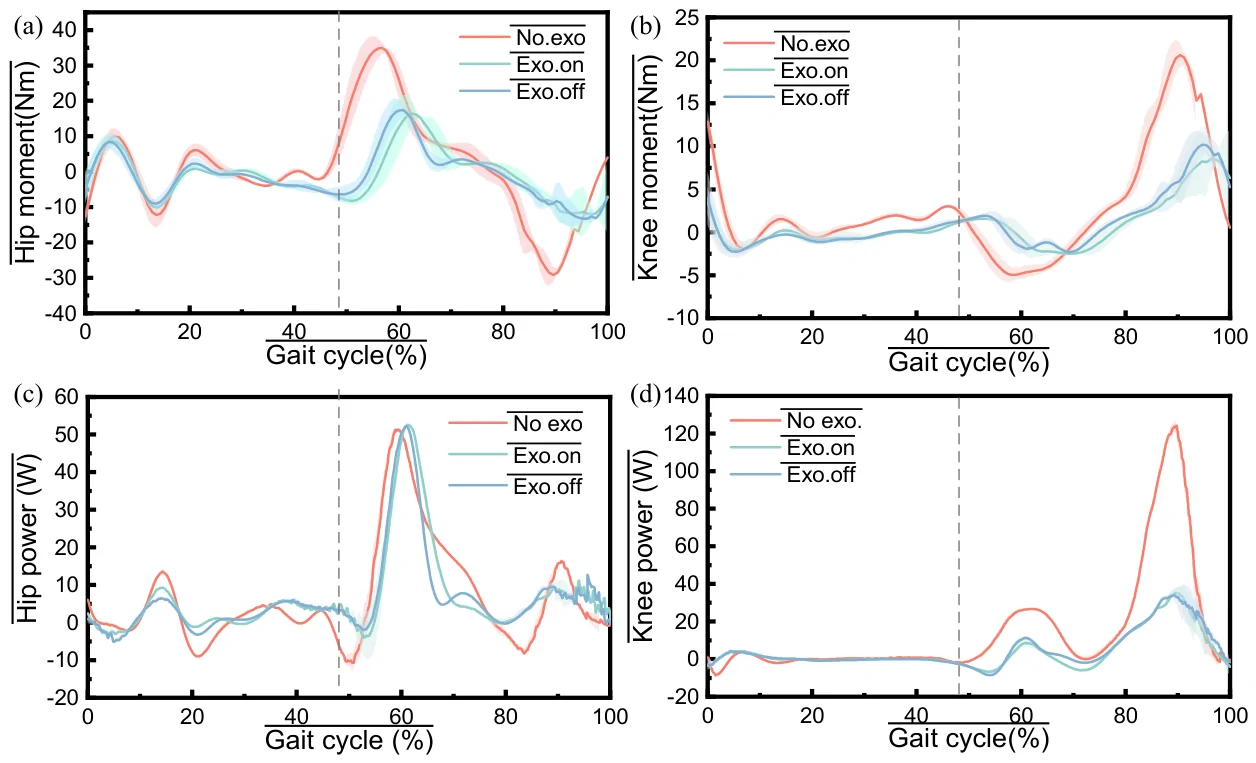
The effect of an unpowered hip exoskeleton on (**a**) hip and (**b**) knee moment, and (**c**) hip and (**d**) knee power.

As shown in Figure 15, it shows the changes in torque and power of the hip and knee joints during the performance test of wearing an unpowered hip joint-assisted exoskeleton. It can be seen from Figure 15a,b that in the “No exo.” state, the range of flexion and extension torque in the sagittal plane of the hip and knee joints is about 64.1 Nm and 25.6 Nm, respectively. In the “Exo. on” state, the range of flexion and extension torque in the sagittal plane of the hip and knee joints is about 28.2 Nm and 12.5 Nm, respectively, which is less than (*P_hip_* = 0.001, *P_knee_* < 0.001) that in the “No exo.” state. In the “No exo.” state, the maximum flexion and extension torque in the sagittal plane of the hip and knee were about 34.9 Nm and 20.6 Nm, respectively. In the “Exo. on” state, the maximum flexion and extension torque in the sagittal plane of the hip and knee joints were about 16.4 Nm and 10.0 Nm, respectively, which were reduced by 53.0% (*p* < 0.001) and 51.5% (*p* < 0.001) compared with the “No exo.” state. As shown in Figure 15c,d, it can be seen that the power range of the hip and knee joints in the “No exo.” state is about 62.0 W and 132.7 W, respectively. In the “Exo. on” state, the power of the hip and knee joints is about 56.6 W and 42.0 W, respectively, which is reduced by 8.7% (*p* < 0.001) and 68.3% (*p* = 0.003) compared with “No exo.”. The experimental results show that the peak value of hip and knee joint torque and power of “Exo. on” is less than that of “No exo.” which is caused by the assist performance of the exoskeleton. Compared with the energy storage phase of the exoskeleton, the torque and power of the hip and knee joint in the “Exo. on” state in the energy release phase is significantly lower than those in the “No exo.” state, which greatly reduces the energy of the positive work phase of the human lower limb, and compensates part of the positive work energy to the negative work phase of the lower limb, so as to effectively achieve the purpose of walking assistance.

### 4.2. Results and Analysis of Sufficient Pressure

For each walking condition, dynamic plantar pressure measurements collected during the stance period—from the initial contact of the right heel with the ground to the subsequent toe-off event of the same foot—were selected for analysis. After normalization of the acquired data, the displacement length of the plantar center of pressure and the maximum plantar pressure were quantitatively compared among the three gait conditions.

**Figure 16:**
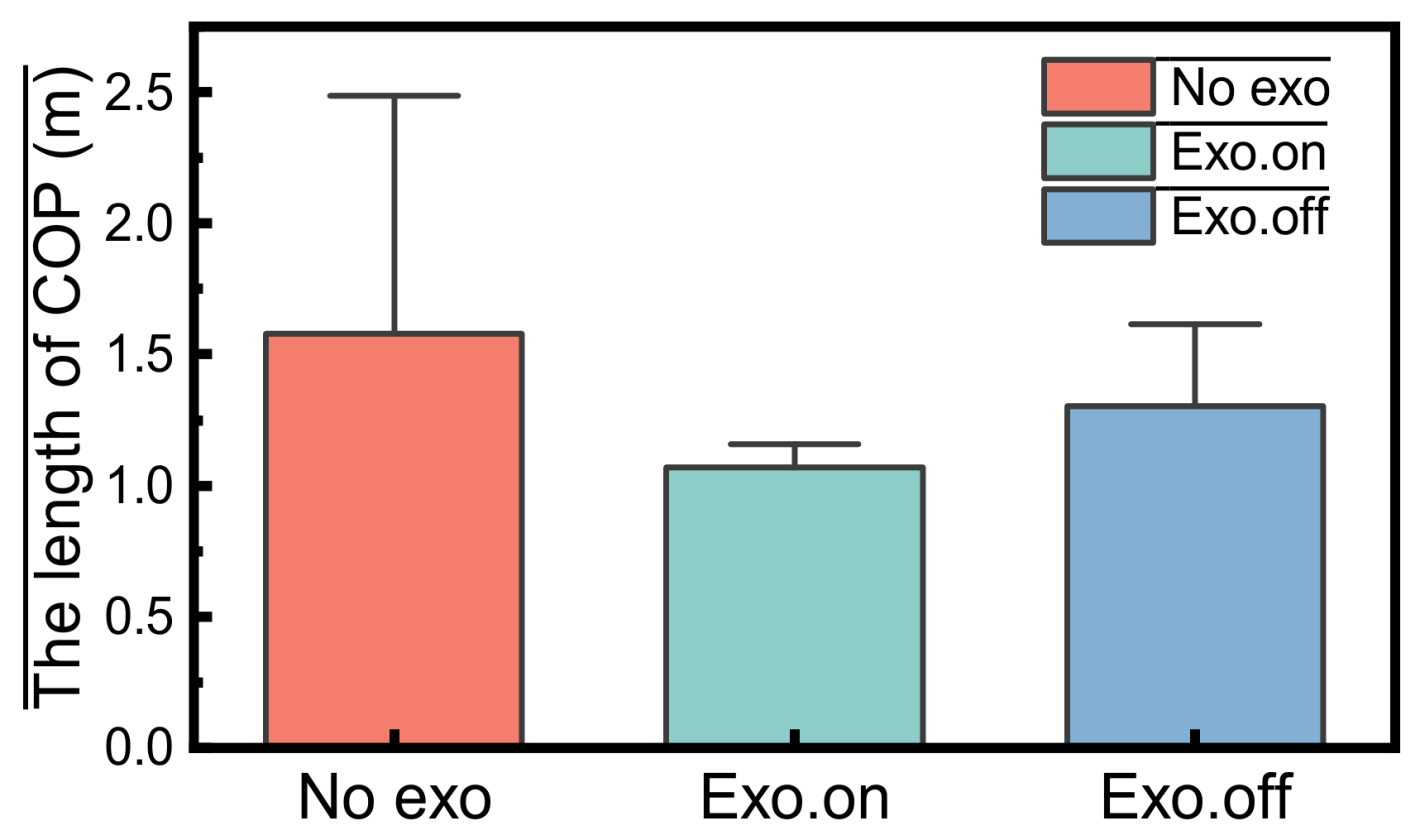
The effect of unpowered hip exoskeleton on the length of plantar pressure center.

As shown in Figure 16, it is a box diagram of the movement length of the plantar pressure center of the experimental object in the “No exo.”, “Exo. on” and “Exo.off” states. In the standing stage of the right leg, compared with the movement length of the plantar pressure center in the “No exo.” state, the movement length in the “Exo. on” state was significantly reduced. The experimental results show that wearing an unpowered hip-assisted exoskeleton can help reduce the time of standing on one leg during walking. Compared with the “Exo. off” state, the movement length of the plantar pressure center in the “Exo. on” state is reduced by 32.3% (*n* = 39 groups; one-way repeated-measures ANOVA; F = 6.982, *p* = 0.003; LSD post hoc analysis: *p* = 0.027). Therefore, the extra mass of the exoskeleton has no significant effect on the movement length of the plantar pressure center in IPS.

**Figure 17:**
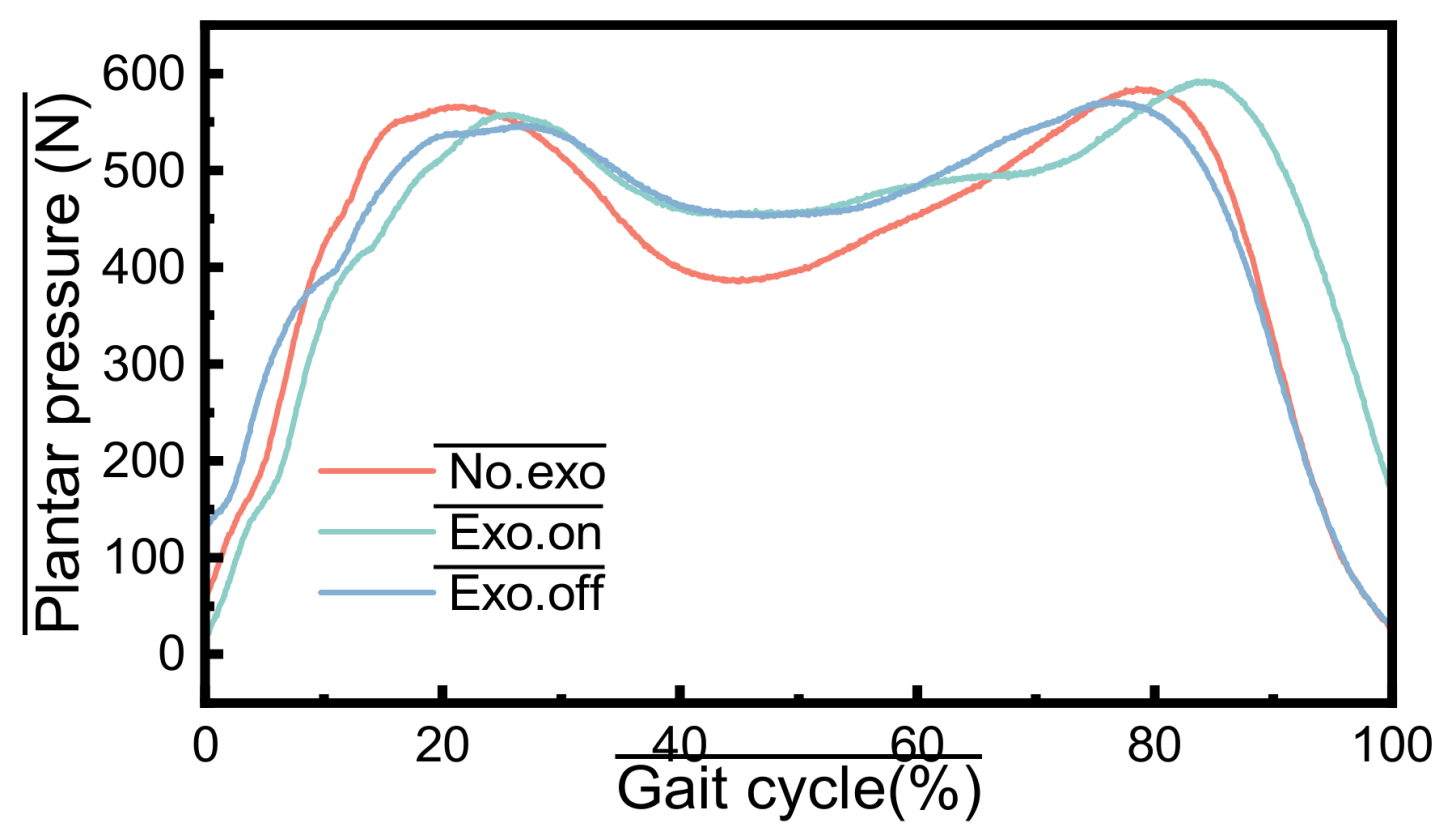
The effect of an unpowered hip exoskeleton on plantar pressure.

As shown in Figure 17, the maximum plantar pressure of healthy subjects in the “Exo. on” state is greater than that in the “No exo.” state (*p* = 0.046). It can be seen that at the trough of the maximum plantar pressure curve, the maximum plantar pressure in the “No exo.” state is far lower than that in the “Exo. on” and “Exo. off” states. The experimental results show that when the exoskeleton is worn with or without assistance, the pressure on the sole of the foot will increase when the exoskeleton is worn because the human body is additionally wearing the assisted exoskeleton. During the exoskeleton energy storage stage, on the one hand, the human body resists the release of additional energy from the energy storage device, and on the other hand, the extra mass of the exoskeleton generates inertial force.

### 4.3. Physiological Results and Analysis

**Figure 18:**
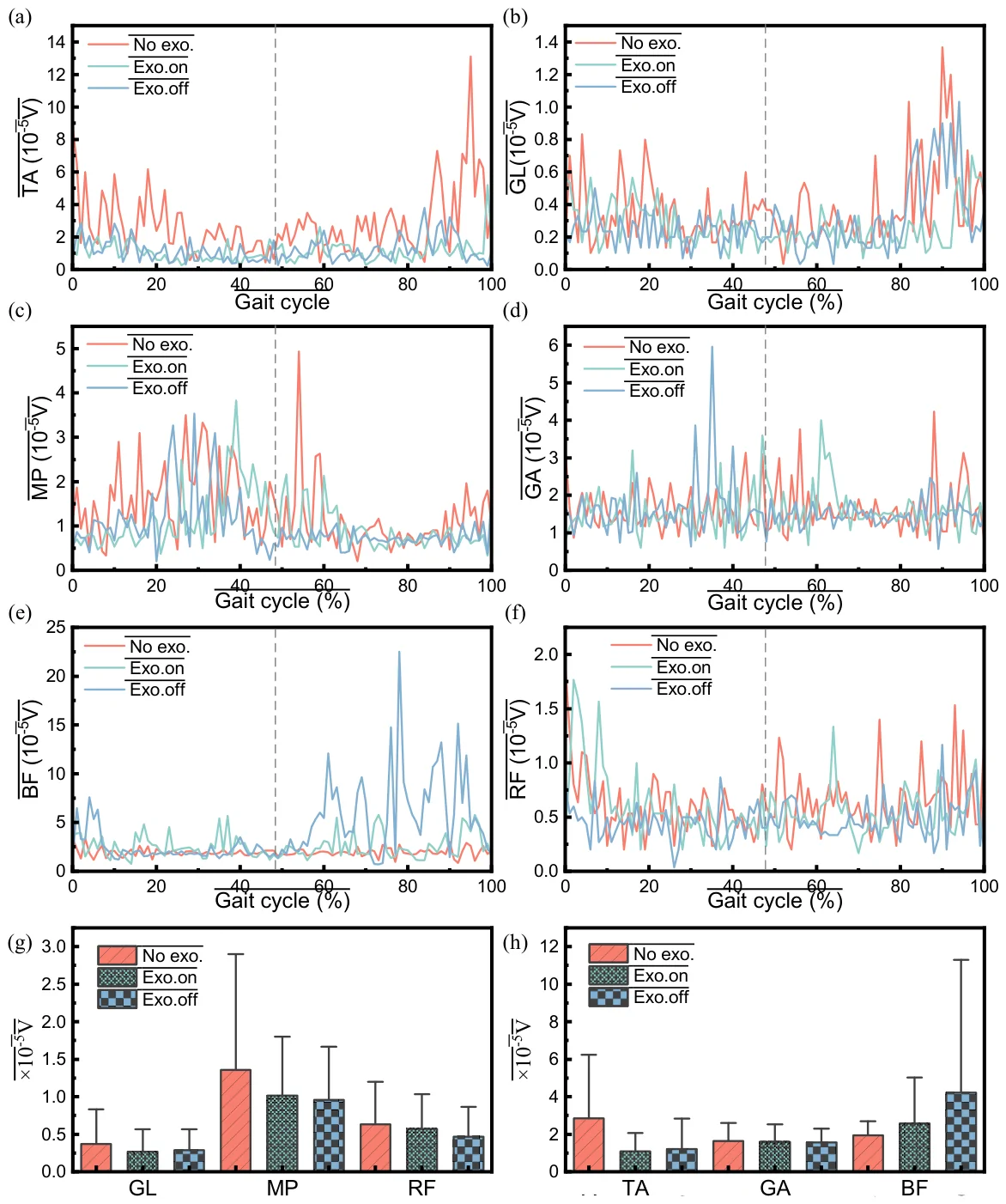
The effect of unpowered hip exoskeleton on lower limb muscles: (**a**) Tibialis anterior; (**b**) Gluteus maximus; (**c**) Medial peroneal; (**d**) Gastrocnemius; (**e**) Biceps femoris; (**f**) Rectus femoris; (**g**) and (**h**) are the comparisons of all muscle activation levels.

In the process of lower limb movement, gluteal muscles, anterior thigh muscles and posterior thigh muscles mainly control the flexion and extension of hip and knee joints. Therefore, GL, RF and BF muscles were selected for EMG signal monitoring in this experiment. The posterior leg muscle group, the anterior leg muscle group and the lateral leg muscle group mainly control ankle plantar flexion, dorsiflexion and foot varus and varus movement. Considering whether wearing an exoskeleton will affect the activity of the end of the lower limb, the EMG signals of MP, Ta and GA muscles were monitored simultaneously in this experiment. The surface EMG signals of six lower limb muscles during exercise were collected, and the results of “No exo.”, “Exo. on” and “Exo. off” were compared and analyzed, as shown in Figure 18.

It can be seen from Figure 18 that from the end of the right leg support phase to the initial stage of the right leg re-support phase, this phase is during the release of the hip-assisted exoskeleton energy, while providing forward auxiliary force to the lower limbs. The EMG signals of all muscles except the RF muscle in the “Exo. on” state are lower than those in the “No exo.” state, indicating that the assisted exoskeleton has played a certain role in the assist phase. It can be seen from Figure 18a–c,f–h that in a single gait cycle, when “No exo.” is used, the average EMG signals of TA, GL, MP and RF muscles in the whole gait cycle are about 2.85/0.37/1.36/0.63 × 10^−5^ V, respectively. When “Exo. on” is used, the average EMG signals of TA, GL, MP and RF muscles are about 1.09/0.27/1.02/0.57 × 10^−5^ V respectively, which are reduced by about 61.7% (LSD post hoc analysis: *p* = 0.003), 28.4% (LSD post hoc analysis: *p* < 0.01), 25.1% (LSD post hoc analysis: *p* < 0.01) and 10.1% (LSD post hoc analysis: *p* < 0.05) respectively compared with “Exo. off”, indicating that wearing exoskeleton can effectively reduce the activity of TA, GL, MP and RF muscles.

As shown in Figure 18d,h, the average EMG signal of the GA muscle is about 1.64/1.59/1.57 × 10^−5^ V under the conditions of “No exo.”, “Exo. on” and “Exo. off”, respectively. Since the GA mainly controls ankle plantar flexion and foot valgus, the experimental results show that the EMG activity of the gastrocnemius muscle is not affected by wearing the exoskeleton.

As shown in Figure 18e,h, the average EMG signal of the BF muscle is about 1.95/2.57/4.22 × 10^−5^ V under the conditions of “No exo.”, “Exo. on” and “Exo. off”. Because BF belongs to the posterior thigh muscle group, it mainly controls the hip extension and knee flexion in the sagittal plane, thus driving the hip exoskeleton to do negative work. Therefore, the EMG signal of BF in the “Exo. on” state is higher than that in the “No exo.” state. On the other hand, because of the extra mass of the wearable exoskeleton tied to the thigh and calf, BF does more negative work to balance the extra mass of the exoskeleton. Compared with “Exo. off”, the EMG signal of BF in “Exo. on” is reduced by 39.0% (LSD post hoc analysis: *p* < 0.001). This result proves that without considering the impact of additional mass, the hip-assisted exoskeleton can reduce the overall lower limb muscle activity, so as to achieve the purpose of reducing walking energy consumption.

**Figure 19:**
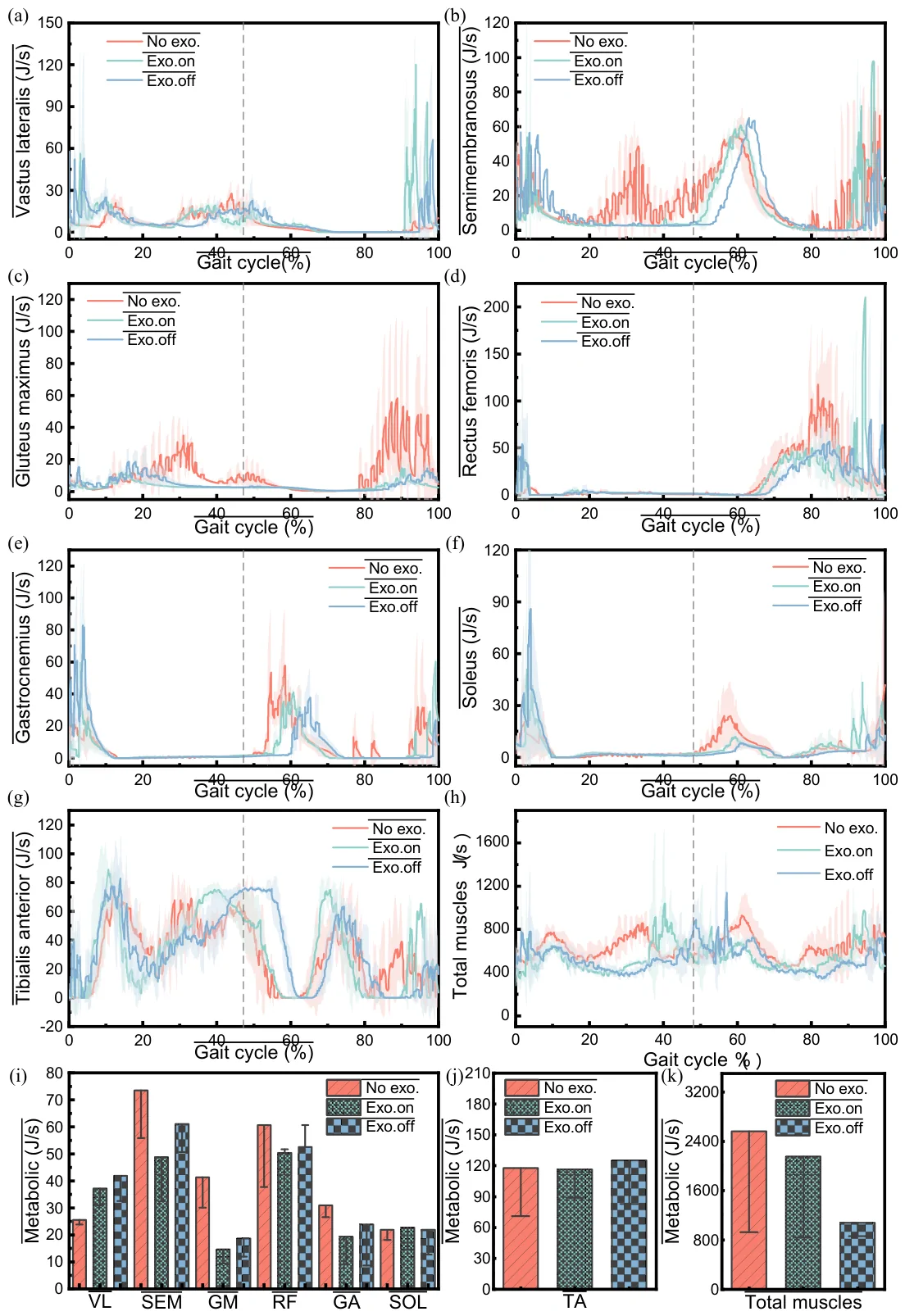
The effect of unpowered hip exoskeleton on metabolic energy consumption of lower limb muscles: (**a**) Lateral femoral; (**b**) Semimembranosus; (**c**) Gluteus maximus; (**d**) Rectus femoris; (**e**) Gastrocnemius; (**f**) Soleus; (**g**) Tibial anterior; (**h**) all muscles; (**i**–**k**) are comparisons of muscle metabolic energy consumption.

By collecting the spatio-temporal data of gait during exercise, combined with OpenSim dynamics analysis, metabolic probes were added to the muscle–bone model to obtain the characteristics of lower-limb muscle metabolism and total metabolism during the gait cycle. It can be seen from Figure 19b–e,i that when “No exo.”, the metabolic energy of hemi-membranous muscle, gluteus maximus muscle, rectus femoris muscle and gastrocnemius muscle in the whole gait cycle is 18.4/10.4/15.2/18.4 J/s, respectively. When in the “Exo. on” state, the metabolic energy of semi-membranous muscle, gluteus maximus muscle, rectus femoris muscle and gastrocnemius muscle was 12.2/3.7/12.6/4.9 J/s, respectively, which was reduced by about 33.7% (*p* < 0.01), 64.4% (*p* < 0.001), 17.1% (*p* < 0.05) and 73.4% (*p* < 0.001) compared with the “No exo.” state.

According to Figure 19a,f,g,i,j, the metabolic energy of the lateral femoral muscle, soleus muscle and anterior tibial muscle in the “No exo.” state is 6.4/5.5/29.6 J/s, respectively. When in the “Exo. on” state, the metabolic energy of lateral femoral muscle, soleus muscle and anterior tibial muscle were 9.3/5.7/29.1 J/s, respectively, which increased by about 45.3% (LSD post hoc analysis: *p* < 0.01,) and 3.6% (LSD post hoc analysis: *p* = 0.920) compared with the “No exo.” state, while the metabolic energy of anterior tibial muscle was almost unchanged. However, when in the “Exo. off” state, the metabolic energy of the tibialis anterior muscle was 31.4% LSD post hoc analysis: *p* < 0.05), and the metabolic energy in the “Exo. on” state was 7.3% (LSD post hoc analysis: *p* < 0.05) lower than that in the “Exo. off” state. It can be seen from Figure 19h,k that for the total metabolic energy consumption of all muscles in “No exo.” and “Exo. on” states, the metabolic energy consumption per unit time is 641.8 J/s and 539.2 J/s respectively, that is, the total metabolic energy consumption is reduced by 16.0% (LSD post hoc analysis: *p* < 0.01) after wearing unpowered hip exoskeleton, which further illustrates that the auxiliary exoskeleton developed by wearing can reduce energy consumption.

To sum up, the three test tasks established in this chapter are closely related to the performance test of the wearable power-assisted exoskeleton of the unpowered lower limb. “No exo.” task is to test the experimental object without wearing the exoskeleton. This experimental task usually needs only one action to complete, such as walking on a horizontal or inclined treadmill. “Exo. on” task is the test of wearing power-assisted exoskeleton, which is consistent with the test action of the previous task. The “Exo. off” task is to release the energy storage components so that they do not work on the basis of the second task, and repeat the same action for testing. The main purpose of adding this task is to eliminate the influence of the extra mass of the exoskeleton on its overall power-assisted performance after wearing, which can be used to guide the improved design of the quality of the exoskeleton. If all collected data are checked to be valid, the next stage of evaluation can be started. Based on the above experimental test results, collect, analyze, and process the initial experimental data to obtain PI information. On the basis of meeting the statistical analysis, once all the recorded PIs prove that the tested exoskeleton plays a dynamic auxiliary role in the test process, it finally shows that the basic performance of the exoskeleton equipment meets the standard. In a word, a CE-ULLWE method, which is mainly used in military or industrial fields, can characterize the eight basic Pi of reducing human metabolic energy consumption, delaying fatigue and increasing body speed. It can be used as a standard method for evaluating the performance of a wearable lower limb exoskeleton.

### 4.4. Discussion

To contextualize the performance of the proposed unpowered hip exoskeleton, the experimental results obtained were compared with the results reported in recent studies on passive hip exoskeletons. In terms of metabolic reduction, Zhou et al. [6] reported a 7.2% ± 1.2% reduction during walking using an unpowered hip exoskeleton with a constant torsional spring, while Zhang et al. [30] achieved a 9.14% ± 0.85% reduction by matching the variable stiffness feature of the human hip joint. The total metabolic reduction of 16.0% observed in our study substantially exceeds these reported values, indicating superior assistive efficiency of the proposed design. Regarding joint torque reduction, our exoskeleton reduced the hip and knee joint torque ranges by 56.0% and 51.2%, respectively, whereas Zhang et al. reported only a 10.03% ± 2.91% reduction in the mean hip joint moment [31]. This marked difference suggests that our energy storage and release mechanism provides more effective unloading of the lower limb joints during walking. For joint power, our exoskeleton reduced knee joint power by 68.3%, substantially higher than the 21.78% ± 5.49% peak hip power reduction reported by Zhang et al. [30], demonstrating the strong energy-recycling capability of the proposed mechanism. In terms of muscle activity, the adaptive dynamic energy-matching exoskeleton reported a 49.4% reduction in iEMG of the vastus medialis [28], while our exoskeleton achieved reductions of 61.7% for the tibialis anterior and 39.0% for the biceps femoris, confirming effective muscular unloading across multiple lower limb muscle groups. Overall, the comparative analysis demonstrates that the proposed unpowered hip exoskeleton outperforms existing passive exoskeleton designs in multiple key performance indicators, including metabolic cost reduction, joint torque unloading, joint power reduction, and muscle activity suppression. These advantages can be attributed to the optimized energy storage and release mechanism that better matches the biomechanical characteristics of human gait.

The experimental results presented above demonstrate the effectiveness of the proposed unpowered hip exoskeleton in reducing joint torque, power, and metabolic energy consumption during walking at a self-selected speed. However, a notable limitation of the current design is its lack of adaptability to different walking or running speeds. As shown in the biomechanical simulation results of Section 2.1, the rotational stiffness of the hip joint varies significantly with motion speed—specifically, the hip rotational stiffness at 5 m/s is substantially higher than that at 2 m/s. The current exoskeleton employs a fixed-stiffness elastic element with a manually adjustable preload, which requires the user to manually tune the knob when switching between different locomotion speeds. This manual adjustment is not only inconvenient but also impractical for real-time speed transitions during daily activities.

To address this speed-adaptability challenge, several promising directions can be pursued in future work. First, the development of variable stiffness mechanisms that can automatically adjust the elastic element’s stiffness in response to real-time gait detection would enable the exoskeleton to adapt seamlessly to speed changes. Second, the integration of adaptive control strategies, such as model predictive control or reinforcement learning-based impedance control, could allow the device to continuously optimize its assistance profile based on sensory feedback from inertial measurement units or plantar pressure sensors. Third, and perhaps most intriguingly, brain–computer interface (BCI) technology offers a novel paradigm for exoskeleton control and adaptation. Recent bibliometric analyses have identified BCI as a rapidly growing research frontier in rehabilitation engineering, with neural network-based decoding methods enabling more intuitive and responsive human-robot interaction [32]. By decoding the user’s intended gait speed and movement mode directly from neural signals, a BCI-controlled exoskeleton could provide real-time, subject-specific assistance without requiring manual intervention or predefined gait templates. Although BCI integration remains in its early stages for lower-limb exoskeleton applications, emerging studies on EEG-based gait intention recognition and adaptive BCI frameworks suggest that this direction holds substantial promise for overcoming the speed-adaptability limitation of current unpowered and powered exoskeletons alike.

## 5. Conclusions

The results of this study show that the wearable power-assisted exoskeleton design method for individuals has a good auxiliary power-assisted effect in daily exercise. The comprehensive evaluation method of the unpowered lower extremity wearable exoskeleton (CE-ULLWE) established in this study proposes eight core performance indicators (PI) based on the task to evaluate the effectiveness of performance, which will transform the evaluation of the auxiliary effect of unpowered exoskeleton driven by human skeletal muscle into the generation of biomechanical indicators of human skeletal muscle movement.

Based on the hip-assisted exoskeleton model developed above, gait test, foot pressure test and electromyography test were used to compare the performance in three wearing states: “No exo.”, “Exo. on” and “Exo. off”. Through the wearing exercise test, the function and performance of the exoskeleton were verified and the auxiliary effect was evaluated. The experimental results showed that after wearing the hip exoskeleton, the maximum angular velocity of the hip and knee joint increased by 18.5% and 9.0%, the power of the hip and knee joint decreased by 8.7% and 68.3%, and the movement length of the plantar pressure center decreased by 32.3%. The biomechanical simulation results additionally found that the total metabolic energy consumption decreased by 16.0%. This further verifies that the developed unpowered lower limb assist exoskeleton can reduce human metabolic energy consumption, delay fatigue and increase body speed.

In future research, the study of unpowered exoskeletons will focus on improving their intelligence, lightweighting, energy efficiency, human–machine collaboration, and application expansion, in order to further improve their functionality and applicability, and manufacture exoskeletons that reach industrial and mass production levels, thereby bringing greater convenience and improvement to people’s lives. In addition, by establishing multiple experimental scenarios and conducting real-world experiments, the CE-ULLWE method is continuously improved, with the aim of using it as a unified and widely applicable benchmark for evaluating the performance of wearable lower limb exoskeletons, and promoting wearable robots to meet real market demand.

## Figures and Tables

**Table 1:** Spring parameters.

Name	Value	Name	Value
The height of the spiral line/H (mm)	14	Effective number of laps/n	7
Maximum diameter of the spiral line/D_max_ (mm)	11	Modulus of elasticity/E(GPa)	210
Minimum diameter of spiral line/D_min_ (mm)	8	Shear modulus/G (GPa)	78.8
Wire diameter/d (mm)	0.5	Poisson’s ratio/V	0.3
Intercept/t (mm)	2	Density/(kg m^−3^)	7.86

## Data Availability

The authors declare that all data are incorporated in this paper.
